# Magnetic Induction Sensing of Corrosion on Steel Pipes: Feasibility, Instrument Design and First Test Results

**DOI:** 10.3390/s26051630

**Published:** 2026-03-05

**Authors:** Verena Schifano, Guy Marquis, Pierre-Daniel Matthey, Martin G. Luling, Hamza K. Bennani, Luigi Kassir, Maher Kassir

**Affiliations:** 1 Institut Terre et Environnement de Strasbourg, UMR 7063 CNRS/Université de Strasbourg/ENGEES, 67084 Strasbourg, France; verena.schifano@enerex.fr (V.S.);; 2 Enerex SAS, 67084 Strasbourg, France; 3 LulingTech SAS, 75019 Paris, France; mgluling@gmail.com; 4 Skipper NDT, 75001 Paris, France; h.bennani@skipperndt.com (H.K.B.); l.kassir@skipperndt.com (L.K.); m.kassir@skipperndt.com (M.K.)

**Keywords:** magnetic induction, corrosion, non-invasive techniques

## Abstract

Underground steel pipes are an essential component of the water and energy supply chains, and assessing their damage with standard techniques implies a temporary interruption in their use, often at a high cost to the operators. Evaluating the damage outside of the pipe would minimize these interruptions. In this work, we propose a new approach to investigating corrosion by taking advantage of the reduction in the steel’s magnetic permeability resulting from it. To enhance these variations, the pipe is excited by a static magnetic field produced by a rectangular loop, inducing magnetization in the pipe that will be weaker where corrosion is present. The secondary magnetic fields produced by this magnetization are measured using an array of triaxial magnetic sensors. A desktop study using finite-element modelling confirmed the feasibility of the approach and informed the design of a first prototype. Scans of test pipes over a custom measurement bench show that corroded zones, as well as welding joints, generate significant anomalies with a strong signal-to-noise ratio, easily identified using simple signal processing techniques. These results confirm the viability of this non-invasive magnetostatic methodology.

## 1. Introduction

Localizing subsurface infrastructure from surface measurements has been routinely used by operators for decades. For steel pipes, the strong contrasts between the electrical properties (electrical conductivity σ or magnetic permeability μ) of the steel and the surrounding earth materials are very high: steel is about 1 million times more conductive than its host and thousands of times more magnetically permeable. For this reason, subsurface investigation techniques based on electrical currents and/or electromagnetic fields have traditionally been privileged.

There are several approaches to the electromagnetic (EM) investigation of subsurface pipes. Some are based on classic geophysical techniques such as the analysis of Eddy currents induced in the pipe by primary magnetic fields from a time- or frequency-domain transmitter (e.g., Won et al., 1996 [1]). In this case, the Eddy currents’ amplitude and decay will be controlled by both σ and μ. Others are based on high-frequency EM waves (i.e., ground-penetrating radar [GPR], e.g., Hoarau et al, 2016 [2]) that are sensitive to μ as well as to the dielectric permittivity ϵ. σ also plays a role through the skin effect that controls the depth of penetration of the waves. More recently, Liu et al. (2025) [3] have proposed a rotating array of passive magnetic sensors to detect subsurface pipe, taking advantage of the contrast of magnetic susceptibility χ (a function of μ, cf. Equation (3) below) between the pipe and its host medium to localize the pipe with high accuracy. The above methods have proven their value for delineation of pipes, but their interpretation in terms of damage evaluation remains challenging due in large part to their limited resolution and the non-uniqueness of their solutions.

For damage evaluation, the most popular in-line inspection (ILI) approaches include magnetic flux leakage (MFL), magnetic particle inspection (MPI) and Eddy current testing (ECT). These techniques utilize an invasive pipeline inspection gauge (PIG) deployed inside the pipe, making it unavailable during its investigation. There is a large body of literature on these methods, and we refer the reader to the reviews by Shi et al. (2015) [4], Coramik and Ege (2017) [5], Feng et al. (2022) [6], Huang et al. (2023) [7], Wu et al. (2024) [8] and Liang et al. (2026) [9].

Several authors have proposed non-invasive alternatives to PIGs. Krivoi and Glinka (2018) [10] proposed investigating by injecting AC currents directly into the pipe and measuring the resulting magnetic fields at the surface. The currents are injected over a range of frequencies, from several Hz to a few hundred Hz, to take advantage of the skin effect: high-frequency currents will concentrate on the pipe’s external surface while low-frequency currents will distribute over the pipe’s thickness. Pipe damage will distort the magnetic field lines. Other authors have proposed using low-maintenance and easily available radio-frequency identification (RFID) sensors during the direct- or alternating-current magnetization of the pipeline to detect local μ perturbations. This approach shows great potential: for example, Wu et al. (2024) [11] experimentally found a large increase in resonance frequency shift between a corroded and an intact pipe, making the corrosion localization straightforward.

In this article, we discuss our investigations into the feasibility of investigating subsurface pipe damage using a non-invasive approach based on DC magnetic fields produced by a surface loop. The main difference with the non-invasive methods mentioned above is that since the energy source is an inductive loop, no direct contact with the underground pipe is necessary for its operation.

As a first step, we undertook a numerical simulation of such a system using finite-element modelling. Based on these results, we designed and built a prototype and tested it on a bench custom-built for pipe investigation. We then present the analysis of these measurements with an ad-hoc interpretation workflow.

## 2. Magnetic Induction

We review here briefly the physics of magnetic induction. Consider an object in an ambient DC magnetic field $\vec{H}$. The magnetization $\vec{M}$ of the object induced by $\vec{H}$ is given by

(1) $$\vec{M}=\chi \vec{H}$$

where χ is the magnetic susceptibility. χ characterizes the ability of a material to acquire an induced magnetization.

In SI units, the magnetic induction $\vec{B}$ is defined as

(2) $$\vec{B}=\mu_{0}\left(\vec{H}+\vec{M}\right)=\mu_{0}\left(1+\chi \right)\vec{H}=\mu \vec{H}$$

where $\mu_{0}$ is the vacuum magnetic permeability. So χ is related to the magnetic permeability μ via

(3) $$\chi =\frac{\mu }{\mu_{0}}-1$$

or reversely, the relative magnetic susceptibility $\mu_{r}$ of a material is related to χ by

(4) $$\mu_{r}=\frac{\mu }{\mu_{0}}=\chi +1.$$

Note that even though χ is unitless, its value is related to the units chosen for $\vec{H}$, $\vec{B}$ and $\vec{M}$. SI units will be assumed throughout this paper as follows: $\vec{H}$ and $\vec{M}$ in A/m, $\vec{B}$ in T and $\mu_{0}=4\pi \times 10^{-7}$ H/m. Table 1 shows the magnetic permeability and the electrical conductivity of selected steels. The degree of variability can be quite large.

The magnetization $\vec{M}$, in turn, produces a magnetization current density ${\vec{J}}_{m}=\nabla \times \vec{M}$, itself generating a secondary magnetic field ${\vec{H}}_{m}$ that is added to the ambient field. Therefore, exciting a steel pipe with a magnetostatic field magnetizes the pipe as a function of μ, so measuring the amplitude of the secondary field ${\vec{H}}_{m}$ during the excitation should lead to an evaluation of μ.

But then how is steel’s μ affected by corrosion? There are two major factors that contribute to a decrease in μ caused by corrosion. The first is that corrosion produces materials that have a lower μ than the undamaged steel (e.g., Singh et al., 2004 [13]), so the effective, macroscopic μ will decrease. The second is more related to its experimental determination: the presence of low-μ rust at the surface of a steel pipe will act as a barrier to the primary magnetic field, reducing its magnetic response and hence underestimating its μ.

The secondary field is computed classically by solving $\nabla \times {\vec{H}}_{m}={\vec{J}}_{m}$, in our case with a finite-element scheme. Note that another source of magnetic perturbation is the remanent magnetization ${\vec{M}}_{r}$, which represents the magnetization “frozen” in the steel of the pipe, acquired when the steel cools through its Curie temperature in a magnetic field. Like ${\vec{M}}_{m}$, ${\vec{M}}_{r}$ also produces a magnetic current density ${\vec{J}}_{r}$ and hence a static magnetic field ${\vec{H}}_{r}$. In this paper, we consider that since ${\vec{H}}_{r}$ is static, it is simply part of the ambient field.

## 3. Finite-Element Modeling

We used the COMSOL Multiphysics^®^ software (COMSOL Multiphysics^®^ v. 6.1. www.comsol.com. COMSOL AB, Stockholm, Sweden) to perform the modeling. From hereon, the solutions obtained with this software will be referred to as finite-element (FE) solutions.

### 3.1. Basic Model and Theoretical Validation

**Figure 1:**
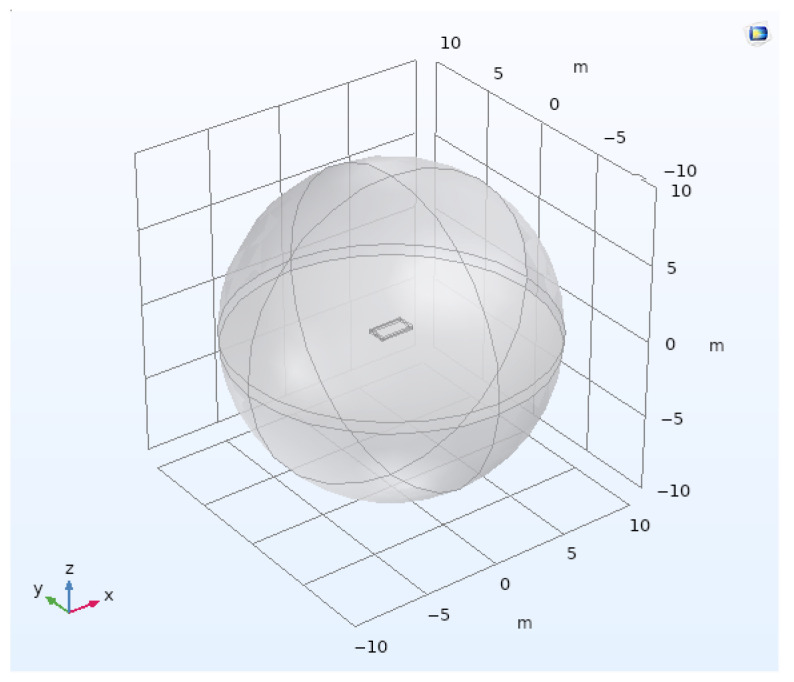
Geometry created on COMSOL containing a centered coil of 2 m × 1 m × 15 cm.

The first step is to define the model geometry: the basic one is shown in Figure 1. To avoid any model edge effects, we have bounded our model by a large sphere of radius 10 m. To model a realistic case of a source at the ground surface, we consider an upper hemisphere with the electrical properties of air and a lower hemisphere with the electrical properties of the earth. We also added a 2 m × 1 m rectangular loop, which will be our current source. All the simulations presented here will have an inductive source (100-turn loop) fed a 10 Ampere AC current at a frequency of 0.1 Hz to get as close as possible to DC conditions. The magnetic field values are in Tesla (and further in nanoTesla).

We chose an AC current for modeling the effect of a DC signal because, in realistic operational conditions, the ambient static magnetic field will strongly bias any DC measurement. To mitigate its effects, we plan to use a half-duty square wave of alternating polarity at 0.1 Hz (Figure 24) that will enable the removal of this bias and allow us to focus on the magnetic induction in the pipe caused by the loop.

We validated our numerical computations before starting elaborate field computations for realistic coil sources and underground pipe geometries. We could have compared the magnetic field produced by a rectangular loop over a half-space estimated using finite elements (FE) for different mesh geometries with an analytic solution (Misakian, 2000 [14]), but there are two main differences to take into account between the FE and analytic solutions. These include (1) the analytic solution being DC (i.e., 0 Hz) while the FE is calculated at 0.1 Hz, as we use an AC computation engine, and (2) the FE loop consisting of 100 turns of wire that are taken into account for its geometry making it 15 cm high, while the analytic solution uses a flat, 2D loop. Nonetheless, both the FE and analytic solutions could be in very good agreement, with some small discrepancies in the cells closer to the source. However, we prefer to step back to the fundamentals: assuming the classical symmetry planes, we expect the $B_{x}$ component to vanish along the plane defined by x = 0, and the same for the $B_{y}$ component along the plane defined by y = 0. Any other values at these locations are caused by numerical errors.

**Figure 2:**
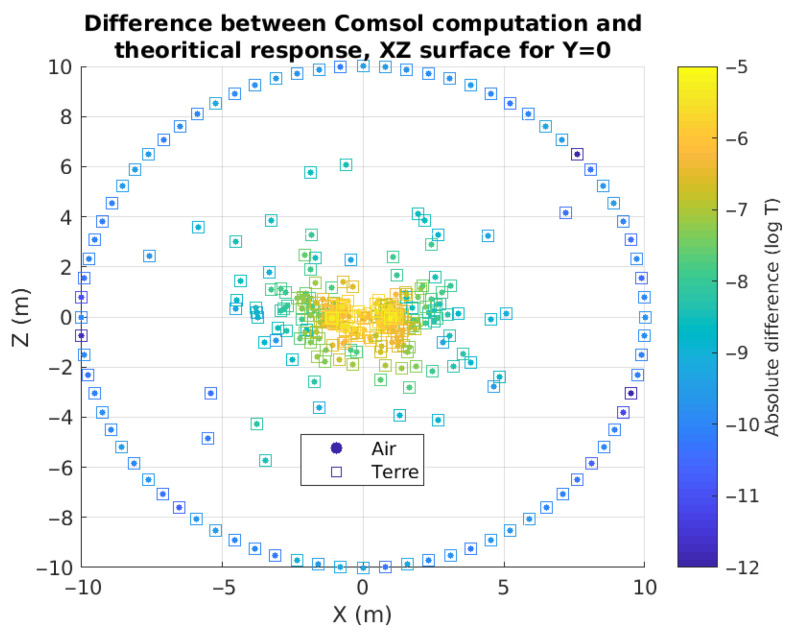
Map of the mesh cell centers, for a simple model, on the plane defined by y = 0. The points represent a modeling with uniform physical properties, and the squares represent a modeling with air–earth properties. The colorization is a function of the COMSOL numerical error on the By component of the magnetic field.

Figure 2 shows the mesh nodes on the XZ plane y = 0 for an extra fine mesh (see mesh characteristics on COMSOL Multiphysics). We computed two models, without modifying the mesh cells: one made entirely of air and one with a half-space of resistivity 10 Ω.m. The mesh has a higher point density around the rectangular loop, as expected for meshing the unique small item of this model. We already see some differences between the computed $B_{y}$ and the theoretical response (as a reminder, this has to be 0 along the XZ plane). The errors increase closer to the source.

**Figure 3:**
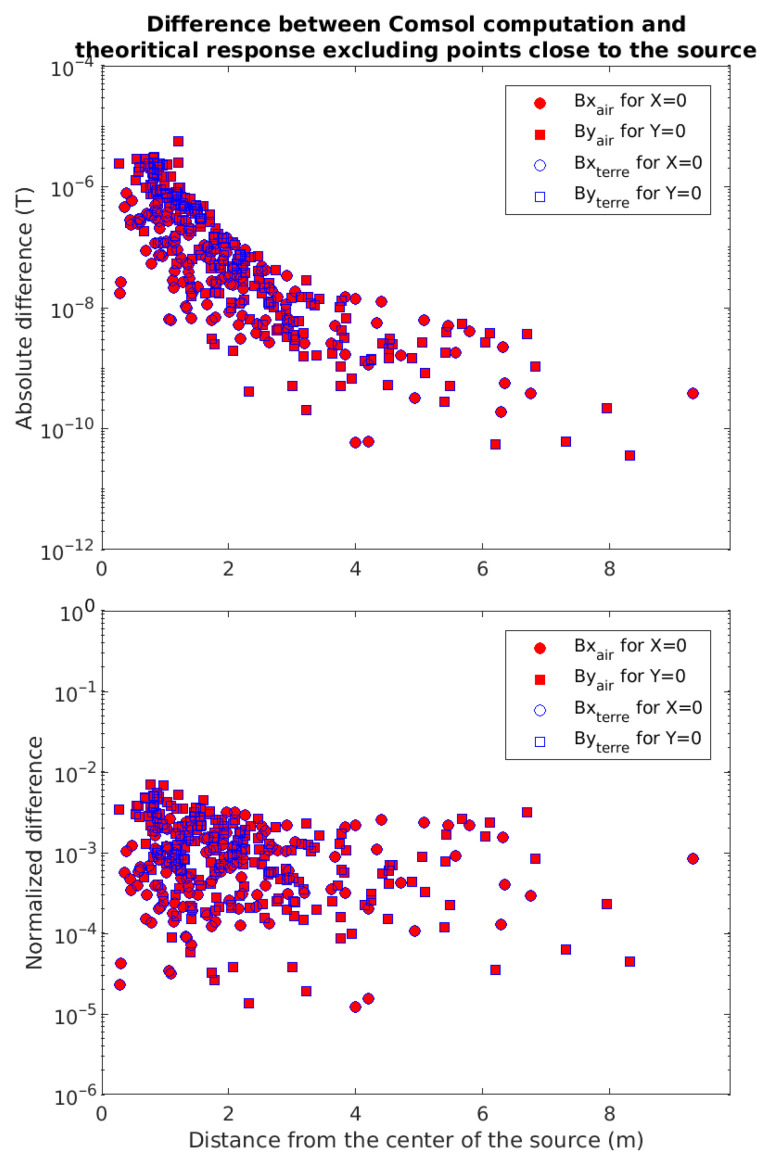
Absolute (**top**) and normalized (**bottom**) FE errors in each cell of the model as a function of the distance from the center of the source, for a homogeneous model (solid red) and a heterogeneous air–earth model (hollow blue). Errors noted on the components Bx (circles) and By (squares) of the magnetic field, respectively on the planes x = 0 and y = 0. For more readability, the cells located within the coil have been masked.

A better way to visualize these differences is to plot them as a function of the distance to the center of the source loop (Figure 3). The differences (top panel) range between a few tenths of nanoTesla to tens of microTesla. Maximal values are close to the loop and drop significantly with distance. Normalized differences (bottom panel) are always less than one percent, and still seem to decrease slightly with distance from the source. We also notice a small decrease in the normalized error towards the center of the rectangular loop, indicating that the maximal error is obtained in the very location of the current loops, where we are not aiming to make any measurement.

These normalized values show that the FE computations are reliable. Moreover, the behavior is the same for both *air* and *earth + air* models, from which we conclude that this error is not related to the physical properties of the model but only to the geometry of the FE mesh chosen.

### 3.2. Construction of the Complete Model

#### 3.2.1. The Pipe Structure

**Figure 4:**
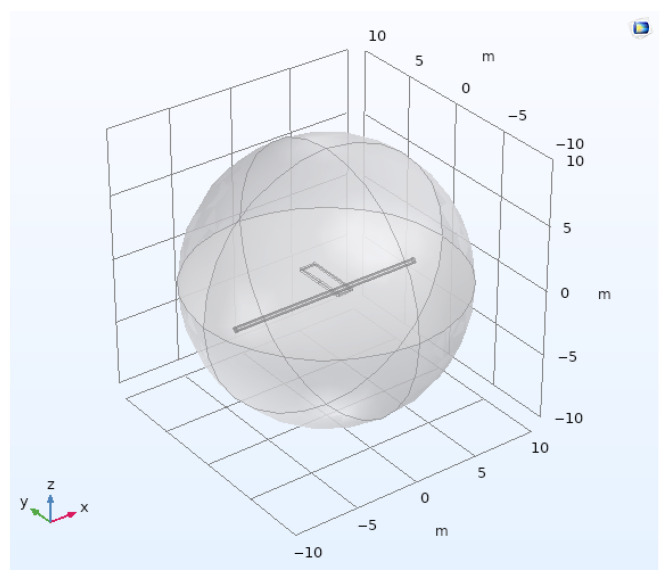
Model created on COMSOL containing a centered coil of 1 m × 4 m × 15 cm, two concentric cylinders, modeling a DN200 casing “buried” 1 m deep with a surface placed at z = 0 m, dividing the model into two hemispheres.

The target pipes are now built from two concentric cylinders of different diameters, with dimensions following industry standards. The vertical distance to the top of the outer cylinder is set at 1 m. The inner cylinder is filled with air, and the space between the two cylinders is filled with steel. The geometry of the model containing a buried pipe structure and a rectangular coil is shown in Figure 4. Table 2 presents the dimensions of the different objects and Table 3 provides the physical properties of the materials.

The first results have highlighted a significant impact of the steel cylinder on the Y and Z components of B and a minor impact on the X component. The Y component is not affected by the loop location; however, the Z component is dominated by it. Hence, we have decided to focus our efforts on the Y component of the magnetic field, which ought to enable better characterization of the targeted object.

**Figure 5:**
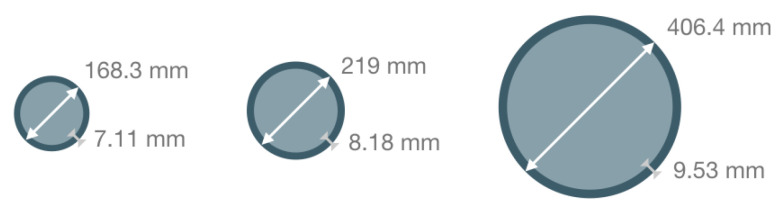
Section of DN 150 (**left**), DN200 (**centre**) and DN400 (**right**) pipes, and their associated diameters and steel thicknesses.

Before going further, we also ran a few tests to evaluate the effect of the type of pipe on the modelling results. We therefore ran the same simple model of a buried pipe with one of the loops (see later) using a small (DN150), a classic (DN200) and a larger (DN400) pipe. The sections of the different pipes, as well as their diameters and steel thicknesses, are shown in Figure 5.

Our modeling showed that there is indeed some sensitivity to the pipe dimensions. Not only do the responses stretch in the Y direction with increasing pipe diameter, but the maximal response amplitudes are 1.2 μT for DN150, 1.7 μT for DN200 and 3.0 μT for DN400. As expected, there is an increase in magnetic field component $B_{y}$ with increasing pipe thickness and increasing pipe diameter: about 30% variation from DN150 to DN200 and almost doubling between DN200 and DN400. Thus, a larger pipe will be easier to locate. Any change in pipe wall thickness due to some form of damage may also be visible. For the next computations, we only consider a DN200 pipe.

#### 3.2.2. Positioning the Source

Our test includes a loop that excites a DN200 pipe at 1 m vertical distance (Figure 4). This source geometry gives better results over its center, which is where we plan to place the sensors. As the errors are smaller away from the edge of the loop, we expect to improve the signal-to-noise ratio (S/N) in the loop center by stretching the loop in the Y direction. The improvement needs have been quantified by modeling.

**Figure 6:**
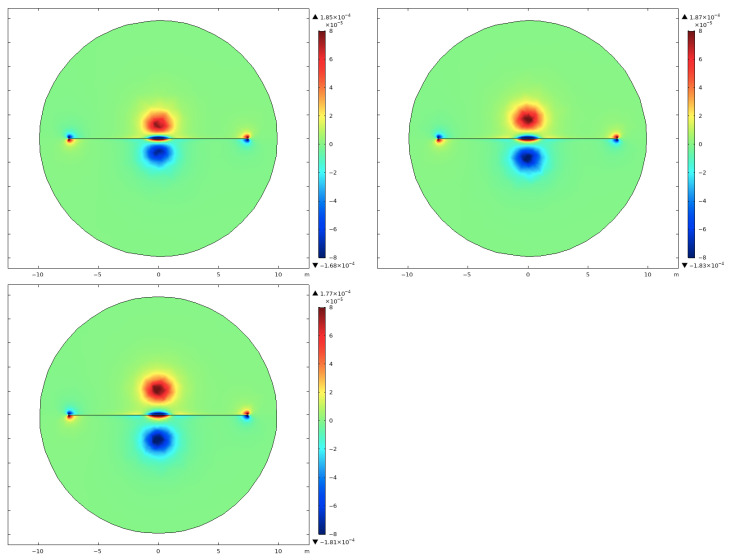
Maps of the By component of the magnetic field computed for a model with a centered coil of of 1 m × 2 m (**top left**), 1 m × 3 m (**top right**) and 1 m × 4 m (**bottom**) upon a DN200 pipe.

Indeed, the position of the source loop relative to the target is key to obtaining a good S/N. To get the best coupling between the source magnetic field and the target, we need to determine the optimal source geometry and position. For this purpose, we have tested several geometries and orientations. One of these tests looks at the length of the loop: 1 m × 2 m, 1 m × 3 m and 1 m × 4 m. Maps of the $B_{y}$ component in the XY plane at z = −1 m for each loop upon an underground pipe, with a constant remanence of $10^{4}$ A/m (see subsequent section about remanence), are shown in Figure 6.

**Figure 7:**
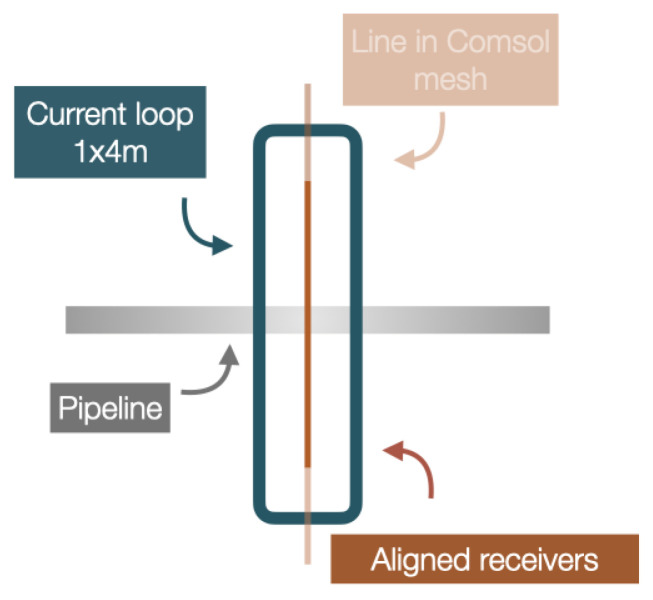
Diagram of the planned acquisition device, considering the source position tests.

As expected, the separation between the strong magnetic fields over the edges of the loop is increased for the greater loop, which should facilitate the detection of the pipe, as there is less risk of an overprint from the source loop. Shifting the loop away from the pipe axis will reduce the effect of these strong magnetic fields even more; however, this shift would also greatly reduce the source-field amplitude at the pipe and hence reduce the induced fields as well. Extending the loop in the direction perpendicular to the pipe ensures that any anomaly recorded by the receivers will have closure, i.e., the field should return to its normal value away from the pipe. Based on these results, Figure 7 shows the layout giving the strongest and unequivocal response, which we will use for the remainder of this paper.

**Figure 8:**
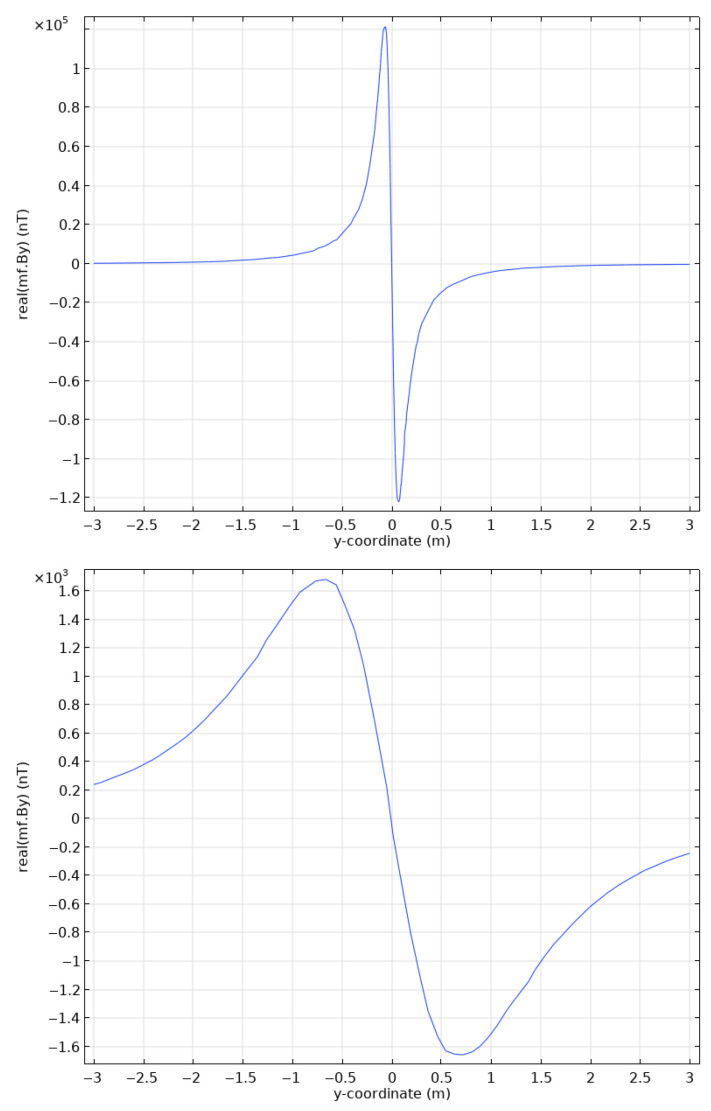
Example of magnetic acquisition at a depth of 1 m (**top**) and on the surface (**bottom**) for a model containing a coil of 4 m × 1 m, serving as a source, placed above a DN200 pipeline buried 1 m deep. Only the real part of $B_{y}$ component is shown here.

In this configuration, the receiver array is located in the center of the current loop, parallel to its long axis (Figure 7). For the remainder of this section, we will present the results of computations only along this line. Figure 8 shows an example of computations for a 4 m × 1 m loop above a DN200 pipe, at the pipe depth (top) and at the ground level, 1 m above (bottom), where we observe the typical horizontal component ($B_{y}$) magnetic anomaly with its inflection point right above the pipe. We also note the filtering effect of moving the instrument away from the target, reducing the peak amplitude from $1.2\times 10^{5}$ nT to $1.7\times 10^{3}$ nT.

### 3.3. Influence of Physical Properties

After evaluating the effects of pipe and source geometry, we now investigate the effects of physical property variations.

First, the pipe remanence magnetization is expected to be approximately $10^{4}$ A/m. This remanence has been implemented to characterize the pipes in the previous tests (Figure 6). The effect of the pipe remanence on the magnetic response is significant and results in two small dipoles at the ends of the pipe. These can be interpreted to first order as point magnetic sources that produce anomalous fields at the ends, with a sharp boundary between the steel pipe on one side and the earth material on the other.

These point-source anomalies are, however, not much of a concern, as their influence is small and in practice, the location of the pipe edges and their joints will be known. In addition, we ran tests on the effect of the remanence orientation. We found that if the remanence is oriented along the Y or Z axes, it is more difficult to separate its effects from those related to magnetic induction on $B_{y}$. A remanence oriented along the X axis has very little effect in the centre of the loop.

**Figure 9:**
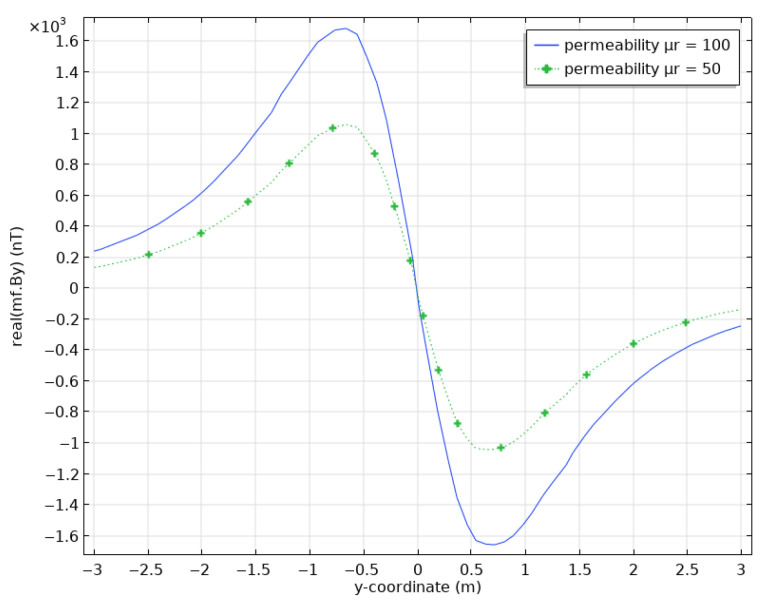
Surface magnetic fields calculated for a steel casing with permeability $\mu_{r}=100$ (blue) and $\mu_{r}=50$ (green dotted line).

We have also studied the influence of the pipe’s magnetic permeability μ on the model responses. As we are interested in using magnetic induction to localize pipe damages, we have run a test with a halved permeability, i.e., $\mu_{r}=50$ to compare with computations using the initial $\mu_{r}=100$. This comparison is shown in Figure 9. The peak value of the magnetic field component $B_{y}$ is reduced by 35%, i.e., a drop from 1700 nT to 1105 nT. This drop is significant and supports magnetostatic measurements to detect pipe damage.

**Figure 10:**
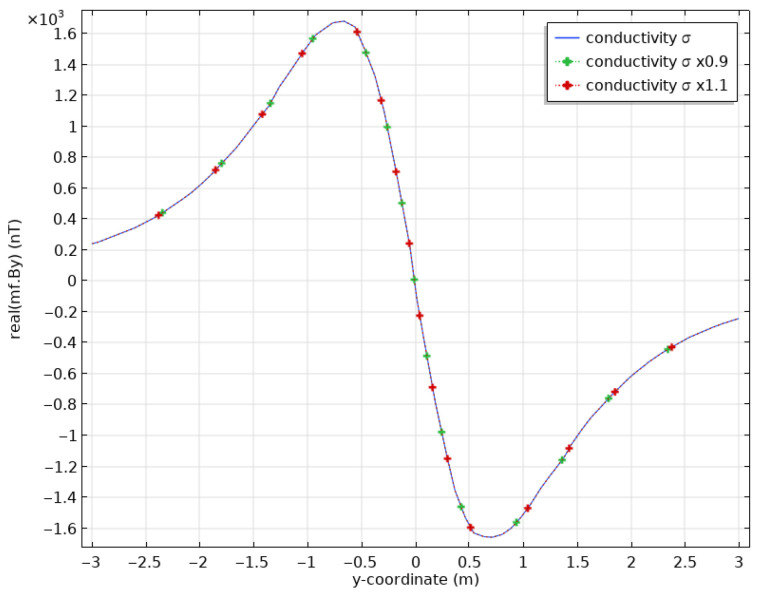
Calculated surface magnetic fields for a steel casing with conductivity $\sigma =10^{6}$ S/m (blue), $\sigma =0.9\times 10^{6}$ S/m (dotted green) and $\sigma =1.1\times 10^{6}$ S/m (dotted red).

Finally, we studied the effect of varying the pipe’s electrical conductivity. We expect its impact to be negligible, but pipe damage, say by oxidation, will have a definite impact on its conductivity. Figure 10 shows a comparison of computed $B_{y}$ between our base-case model and models for which the conductivity has been decreased or increased by 10%. As expected, there is no effect of the pipe’s electrical conductivity on the magnetic induction; any variation in the induced magnetic field must be related to changes in magnetic permeability (i.e., magnetic susceptibility).

### 3.4. Anomalies in the Casing Structure

The models presented so far considered changing the physical properties of a homogeneous pipe. However, damage to pipes is localized, so we will now study anomalies over a small portion of the pipe. We only focus on variations of magnetic permeability that should be visible in the computed magnetic field.

#### 3.4.1. Sections

Our first test will evaluate the effects on a single narrow anomalous section of a pipe, where the section (the damaged zone) of the pipe has a lower magnetic permeability than the intact pipe. To maximize the effect of the anomaly, we will align them with the centre of the transmitter loop. By combining several small sections, we can model the effects of anomalies of any size.

**Figure 11:**
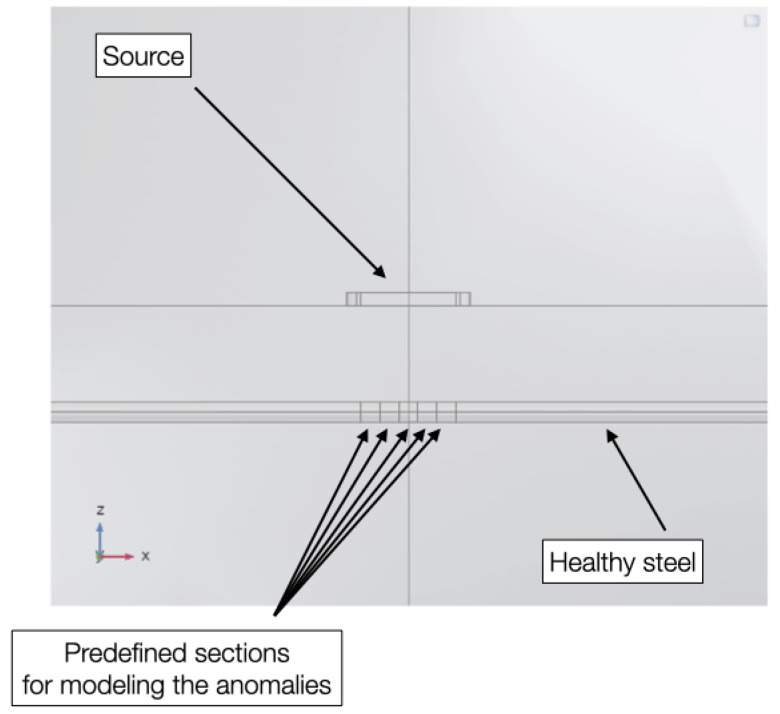
Magnified view of the model showing predefined sections to accommodate anomaly properties.

**Figure 12:**
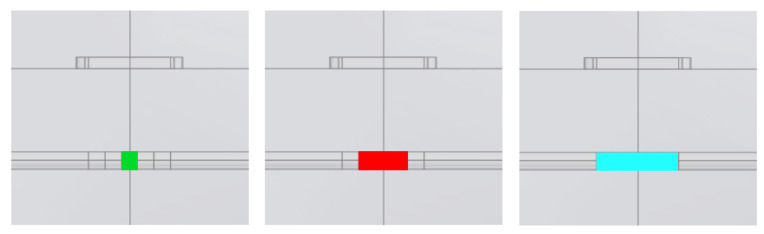
Zoom in the model with an anomaly section 20 cm long (green), 60 cm long (red) and 100 cm long (cyan).

Figure 11 shows a zoom of the model in the XZ plane that highlights the geometry used to compute cross-section anomalies: we sliced the pipe structure along YZ planes at x = ±0.1, ±0.3 and ±0.5 m, resulting in five small pieces, which we combined to obtain the centered anomalous sections. The three anomaly sizes we consider here are shown in Figure 12: in green a 20 cm long section around the model center, in red a 60 cm section and in cyan a 100 cm section.

**Figure 13:**
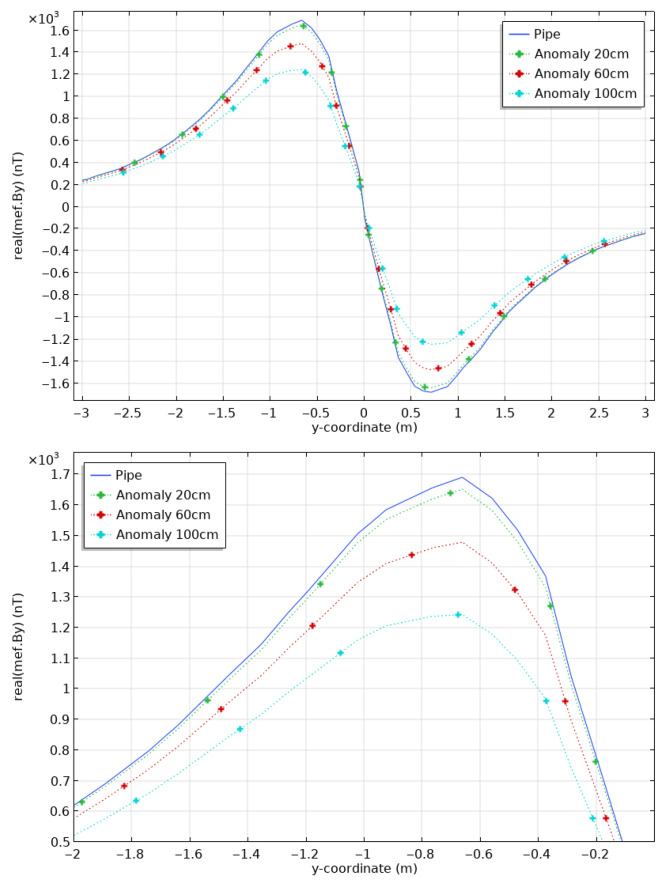
Magnetic fields at the surface (**top**) calculated for a casing of healthy steel (blue), and casings with anomalies of $\mu_{r}/10$, of lengths of 20 cm (green), 60 cm (red) and 100 cm (cyan). Zoom on the peak positive values (**bottom**).

This geometry allows for the modeling of a clean pipe or assigning different physical properties to the sections to simulate anomalies. The comparison of $B_{y}$ computed for the three anomalies, in which the magnetic permeability of the pipe is divided by 10, is shown in Figure 13.

The longer anomalies cause a larger response: our calculations show a difference of up to 2.4% for a 20 cm-long anomaly, 6.6% for a 60 cm-long anomaly and 15.0% for a 100 cm-long anomaly. From now on, we consider that a 20 cm-long anomaly is our target anomaly size as it seems technically possible to detect it with our methodology. Indeed, the difference between the 20 cm anomaly and the fresh casing is of the order of 50 nanoTesla (nT), i.e., well above the resolution levels of commercially-available magnetometers.

**Figure 14:**
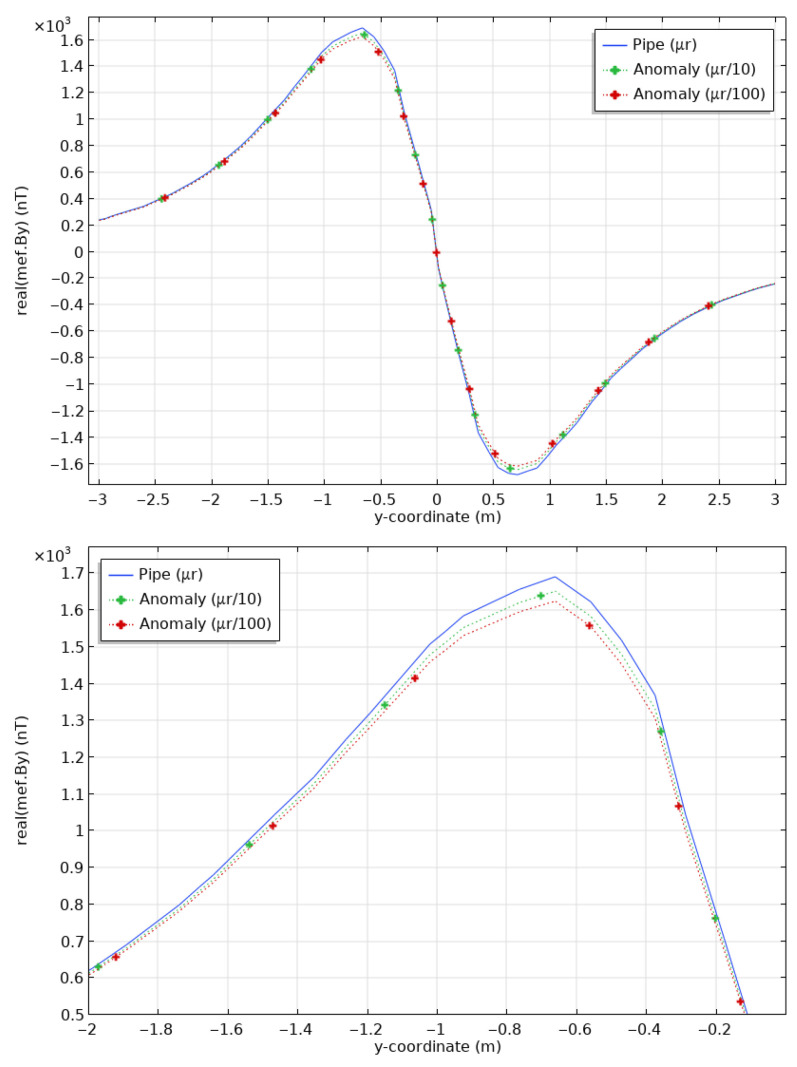
Magnetic fields at the surface (**top**) calculated for a casing of healthy steel (blue), and casings showing an anomaly in section, 20 cm long, with a permeability divided by 10 (green) and by 100 (red). Zoom on the peak positive values (**bottom**).

To illustrate the impact of changes in magnetic permeability for a 20 cm-long anomaly, we computed the responses for anomalies of casing permeability divided by 10 and 100, i.e., $\mu_{r}$ = 10 and 1. Note that the latter case is simply assigning the magnetic permeability of air to the anomaly, which is common practice in numerical modelling. Figure 14 shows $B_{y}$ at the surface. The maximal differences in $B_{y}$ between a clean pipe and a pipe with a 20 cm-long damaged section are 40 nT (2.4%) for $\mu_{r}$ = 10 and 75 nT (4.3%) for $\mu_{r}$ = 1. These values are well above the resolution of commercially-available magnetometers, typically less than 1 nT.

#### 3.4.2. Half-Sections

In reality, it is unlikely that corrosion affects the pipe around its whole circular section, as we assumed in the previous example. Corrosion tends to attack smaller segments and spread around them. To get closer to these more realistic situations, we have considered modelling the effects of a half-section to establish whether the resulting anomalies could be detected. We modelled anomalies localized on a semi-circular section in the upper half of the pipe, still 20 cm in length.

**Figure 15:**
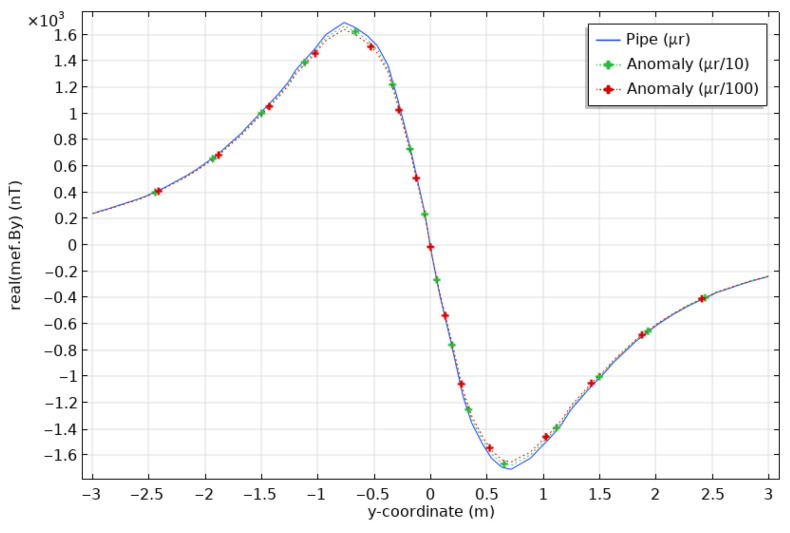

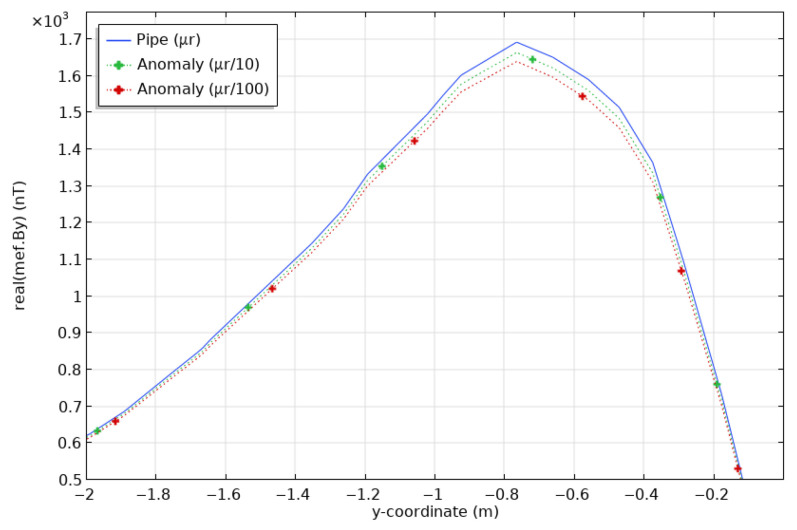
Magnetic fields at the surface (**top**) calculated for a casing of healthy steel (blue), and casings showing an anomaly of upper half-section, 20 cm long, with a permeability divided by 10 (green) and by 100 (red). Zoom on the peak positive values (**bottom**).

**Figure 16:**
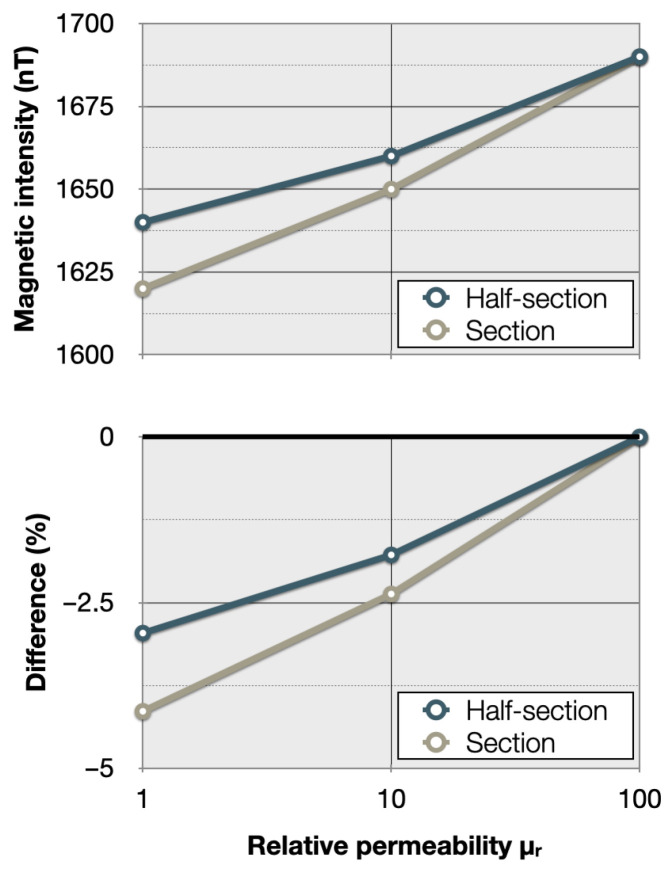
Maximal absolute values (**top**) reached by the magnetic anomaly on the magnetic field curve $B_{y}$ and differences compared to the case of a homogeneous casing of permeability μ = 100 (**bottom**) as a function of permeability magnetic half-section (blue) or section (grey) center of the casing at the surface.

As in Figure 14 for the case of a circular section anomaly, Figure 15 shows $B_{y}$ at the surface for an intact pipe and for a pipe with an anomalous half-section with a permeability $\mu_{r}$ of 10 and 1, thus divided by 10 and 100 according to the permeability of the healthy pipe. These new results are very similar to the ones obtained for circular sections, even though, as expected, the variations are weaker, about 30 to 60 nT. Figure 16 shows the peak $B_{y}$ responses and the percentage differences as a function of μ, for both the section and half-section anomalies. The anomaly remains visible; however, the half-section anomaly shows weaker responses than the full section: at its peak (for the permeability of 1), the section reaches a little more than 4% anomaly compared to the sound casing, while the half-section only reaches a little less than 3%.

#### 3.4.3. Smaller Anomalies

**Figure 17:**
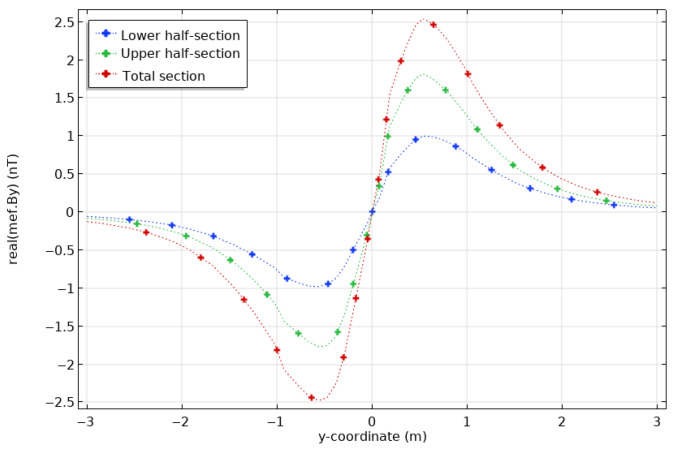
Differences between magnetic fields calculated for casings with an very small anomaly and the sound casing. The 2 cm long anomaly is defined in the lower (blue) and upper (green) half-sections, and in the complete section (red).

We now look at smaller, more localized magnetic susceptibility anomalies to explore the sensitivity limit of the response to ever smaller anomalies. We slice a DN200 pipe along the YZ planes at x = ±0.01 m to obtain a total cross-section of 2 cm. This section is then horizontally subdivided to model half-sections pointing up and down. This study determines the measurement response to the anomaly position. Anomalies have the magnetic permeability of $\mu_{r}=1$ (the maximum we considered until here, which is equivalent to the properties of the surrounding earth); other parameters are the same as for previous examples. Figure 17 shows $B_{y}$ for these small half-sections containing the anomaly. At this stage, the impact of the anomaly is so small that we can no longer consider the difference with the no-pipe model. Instead, we focus on the difference between the models with and without anomalies. First, we see that there still exists a difference between the healthy pipe and the anomalous ones. The differences of a few nanoTesla may be difficult to distinguish above the noise levels in real data. We also see, without surprise, that the weakest response is obtained for the half-section at the lower part of the pipe, and the strongest is obtained for the whole section.

**Figure 18:**
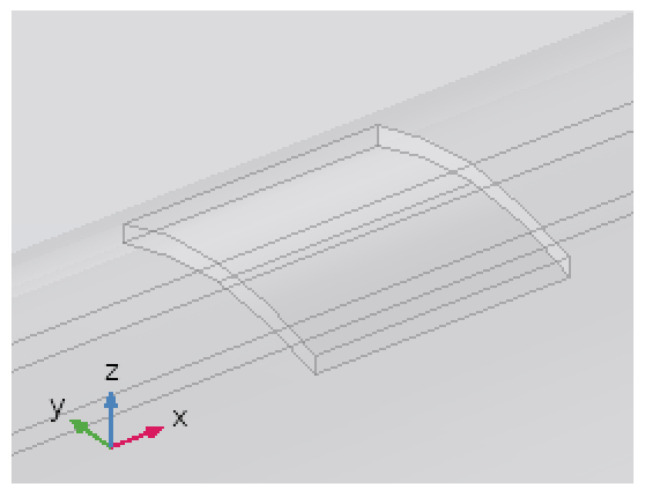
Magnified view of the model consisting of a pipeline with a scale-like anomaly.

**Figure 19:**
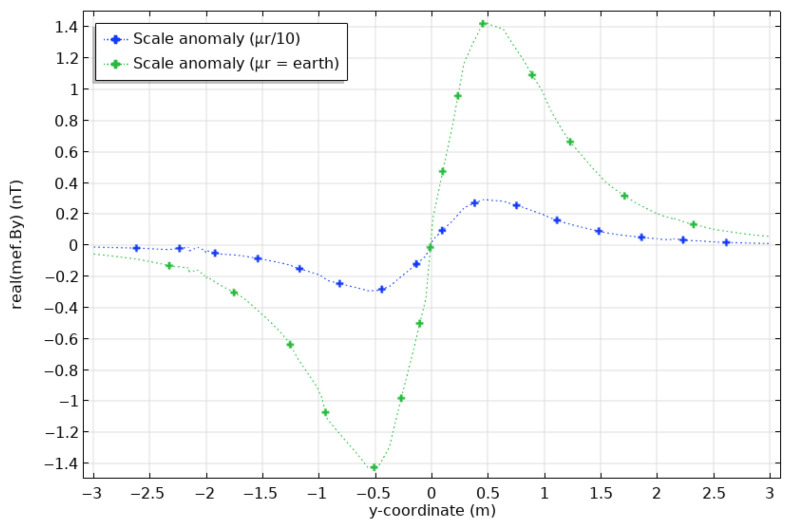
Differences between magnetic fields at the surface calculated for casings with a scale-like anomaly and a casing of sound steel. The scale anomaly has the physical properties of the earth (green) and the relative permeability of the pipe divided by 10 (blue).

On the other hand, we make a final test with an even thinner anomaly. We compute the response for a scale-like anomaly that covers only a part of the thickness of the pipe. We will use an (L × W × H) 80 × 80 × 3 mm anomaly, as shown in Figure 18. The scale was attributed the magnetic permeability of the earth to simulate loss of steel, as well as a permeability of one-tenth of that of intact steel. Modelling results for $B_{y}$ are shown in Figure 19. Clearly, this anomaly is too small to generate a response detectable at the surface. This result put a lower bound on the size of damage-related anomalies that on could detect with the proposed magnetic induction system.

### 3.5. Conclusions from Modelling

To summarize our findings from this desktop feasibility study briefly (see also Table 4 below):

- a probing system based on magnetic induction is sensitive to variations in magnetic susceptibility (e.g., related to corrosion) on a buried pipe;
- the size of the low-permeability volume (i.e., the damaged zone) controls the amplitude of the response, and a larger damaged volume causes a larger magnetic response;
- the position of the damaged portion on the pipe influences the amplitude of the response, and shallower damage causes a greater response;
- except for very small anomalies, anomalous responses can be detected using off-the-shelf magnetic sensors.

We conclude that our numerical modelling computations demonstrate the plausibility of our proposed method for probing underground pipes. These encouraging initial results led us to develop a prototype to test the method in a laboratory experiment.

## 4. Prototype

### 4.1. Design

**Figure 20:**
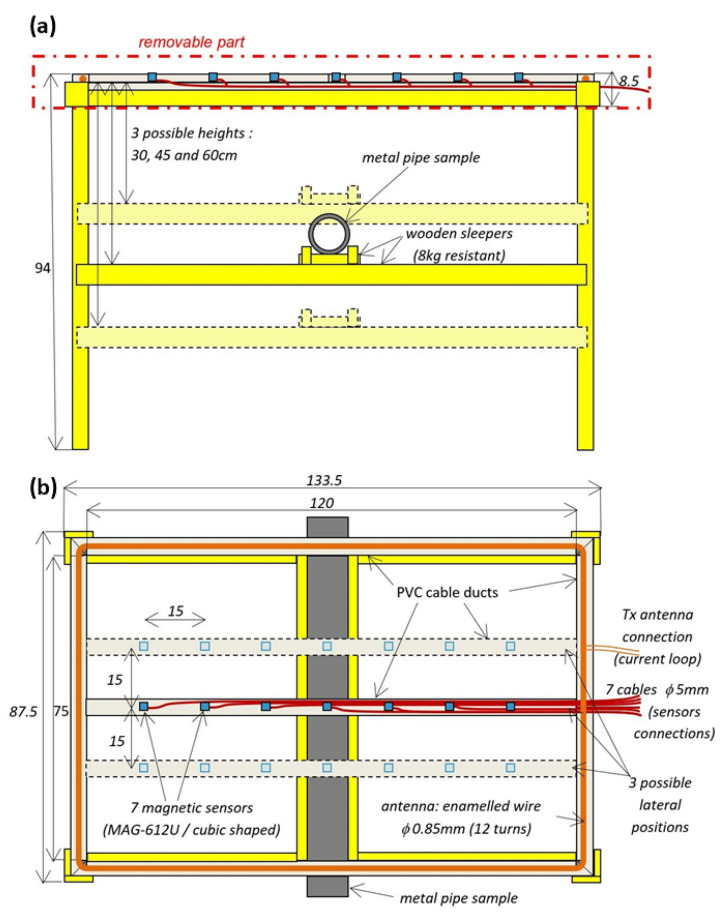
Top- (**a**) and front-view (**b**) of the prototype design, dimensions in centimeters.

Our next development step is a proof-of-concept experiment to be conducted on a purpose-designed bench (see the next Section). For practical reasons, we designed a prototype smaller than the one used in the desktop feasibility study. We have therefore built a prototype with a 120 cm × 75 cm source loop and a central bar holding fluxgate magnetic sensors. Top and side sketches of the prototype are shown in Figure 20.

**Figure 21:**
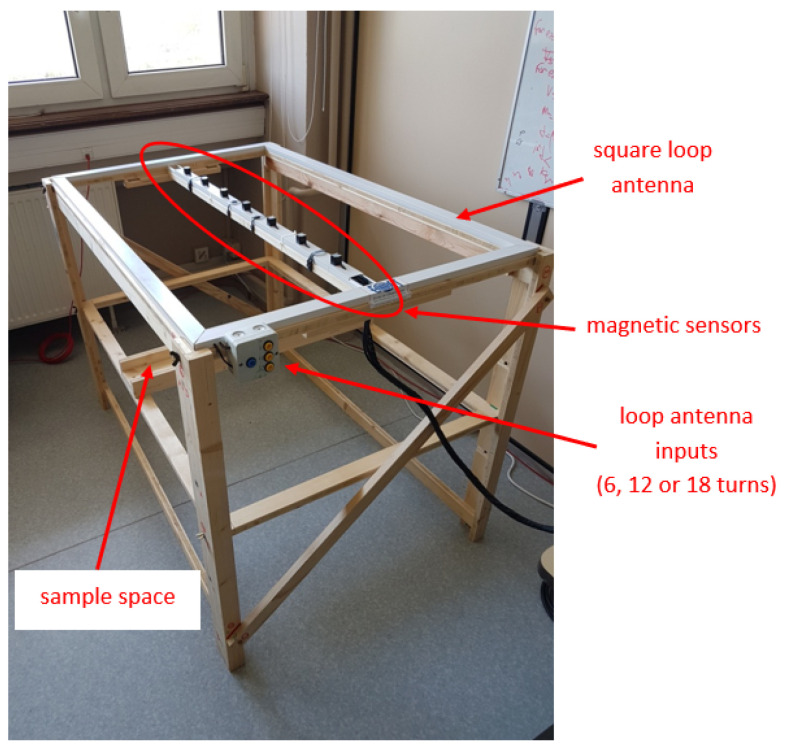
Assembled prototype. Details in text.

In more detail, the prototype (Figure 21) consists of:

- A rectangular-shaped loop source with a number of wire turns chosen by the user: 6,12or18, resulting in a moment of $5.4,10.8\;\mathrm{or}\;16.2\times \;\left(\mathrm{Current}\right)\;{A.m}^{2}$, respectively.
- A bar containing seven Bartington Instruments Ltd. (Witney, UK) MAG-612U^®^ triaxial magnetic sensors 15 cm apart.
- A wooden frame held together by brass screws, to minimize magnetic perturbations.

**Figure 22:**
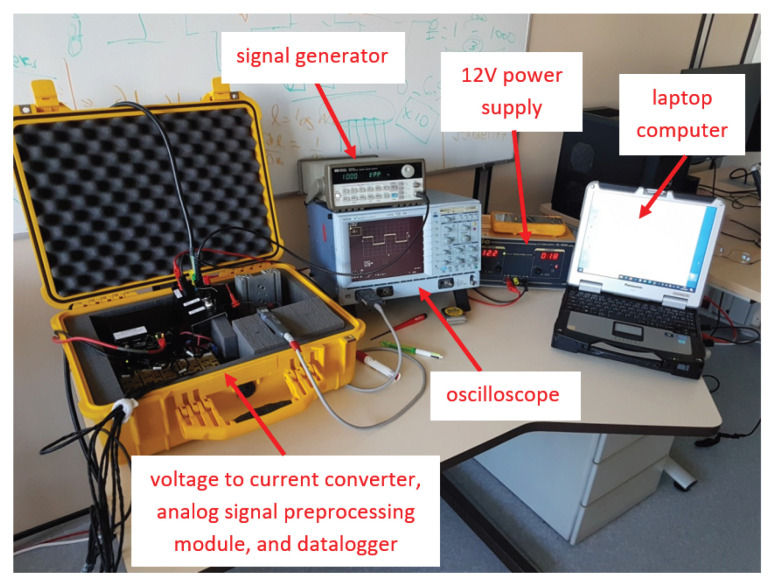
Acquisition system components. Refer to the main text for label descriptions.

**Figure 23:**
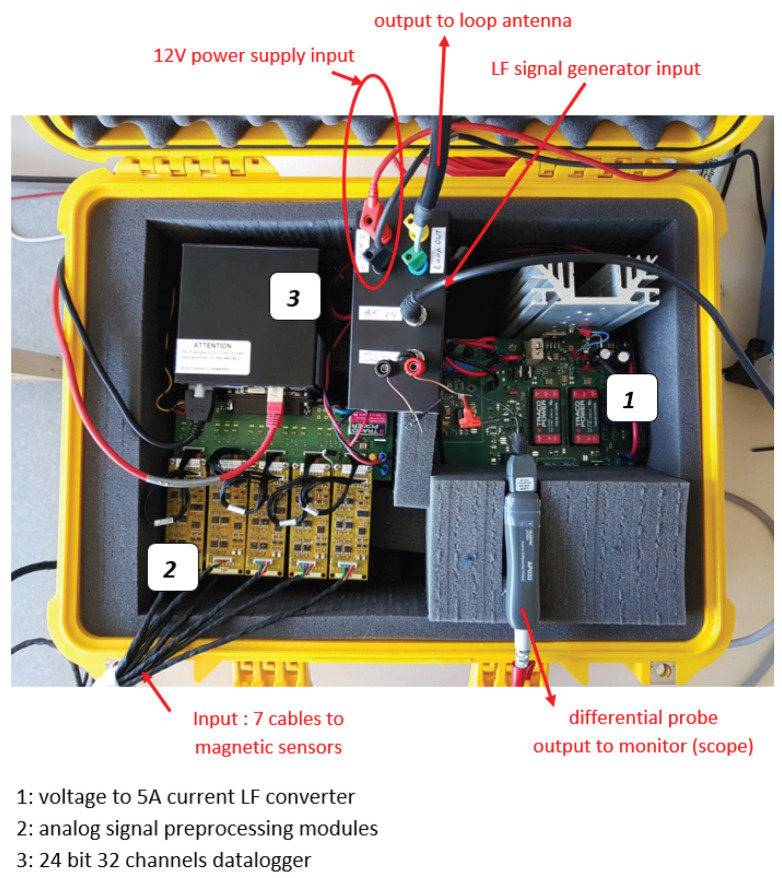
Source and sensor signal conditioning and recording unit.

This prototype is connected to a source current and an acquisition system (cf Figure 22) powered by a single 12V supply. The current source is driven by a Hewlett-Packard (Santa Clara, CA, USA) HP33120A^®^ signal generator and is installed in a case containing the voltage-to-current converter, the sensor amplification and signal conditioning and recording circuit, as described in more detail in Figure 23 below. An oscilloscope is used to monitor the analog signal, and data are transmitted to and the data are transmitted to a 24-bit data logger, which is connected to a laptop computer by an Ethernet port.

The yellow case in Figure 22 and Figure 23 includes a voltage to current converter, a current amplifier, seven sensor signal processing modules (i.e., each sensor has its own power supply and signal conditioning module) and a United Electronic Industries (Norwood, MA, USA) Logger300^®^ with two DNA-AI-217^®^ 16-channel boards. A differential probe is added for real-time visual current inspection on the oscilloscope.

**Figure 24:**
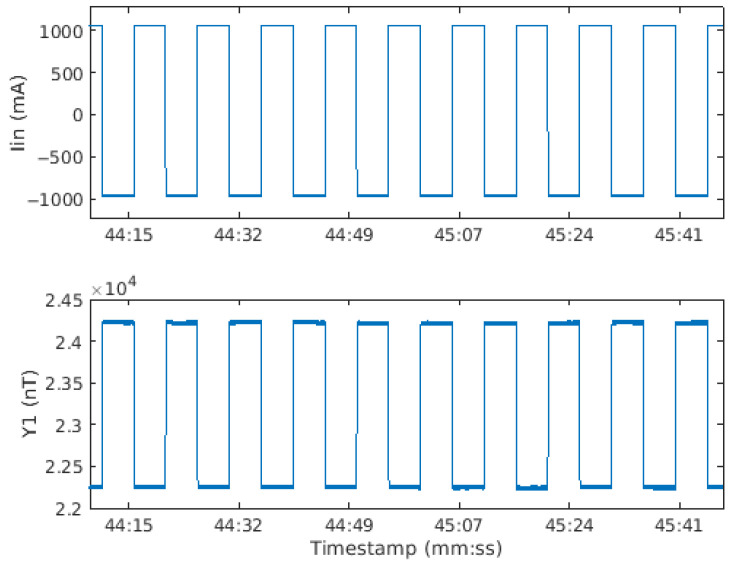
Example of data recorded. Top: source current in mA, bottom: $B_{y}$ in nT for the central sensor.

The source signal is a sequence of alternating positive and negative steps of constant current (cf. Figure 24). This source waveform has the advantage of producing magnetostatic fields in two opposite directions, so DC bias, say due to the earth’s magnetic field, noise or remanence, can be removed. Using a rectangular waveform has the effect of producing Eddy currents when the current drops or rises rapidly. However, Eddy currents in steel pipes are short-lived (less than 1 millisecond, e.g., do Carmo et al., 2016 [15]) and our data are sampled at 1000 Hz, so only a very small fraction (if any) of the recorded magnetic fields are related to Eddy currents. For the experiments described here, we chose a square-wave frequency of 100 mHz or a 10-s period.

We measured the source current with a Hall effect probe integrated into the amplification circuit. The source current and the signals from the 3-component sensors are recorded on an SD card by the datalogger, so overall 22 channels are recorded at a sample frequency of 1000 Hz. After each measurement run, data are transferred to the PC via an Ethernet port for further processing and interpretation.

### 4.2. Data Processing

To illustrate the acquisition, one minute of recordings is presented in Figure 24. The top panel shows the current waveform with an amplitude of ±1000 mA, and the bottom panel shows the Y-component of the central magnetic sensor. As the fluxgate sensor measures $\vec{B}$ (and not $\partial \vec{B}/\partial t$ like a coil sensor would do), the earth’s magnetic field and other surrounding magnetic perturbations add a DC shift to the magnetic measurement, for example, shown here of about 23,200 nT. This DC shift will be removed by processing, and the amplitude of the Y-component response to the prototype’s source field is about 2000 nT peak-to-peak. The maximal value of the magnetic induction that can be measured by a sensor (component x, y, or z) without saturation is ±90,000 nT.

To extract significant information from these recordings, we applied the following processing steps:

1. manual selection of the start of the first whole current box and the end of the last one—the usable time series are in between;
2. extract the same time interval for all the magnetic components;
3. obtain peak-to-peak values for all boxes, for all magnetic components and the source current;
4. average peak-to-peak for each component and the source current, i.e., twice the response;
5. plot responses as a function of the sensor.

Of course, there are many other ways to extract parameters from these time series, but we found this simple approach to be robust. More sophisticated transfer-function-estimation methods might be necessary when the data quality is poor, or noise levels are very high and/or unstable (i.e., shifts in the magnetic measurements cannot be considered DC).

**Figure 25:**
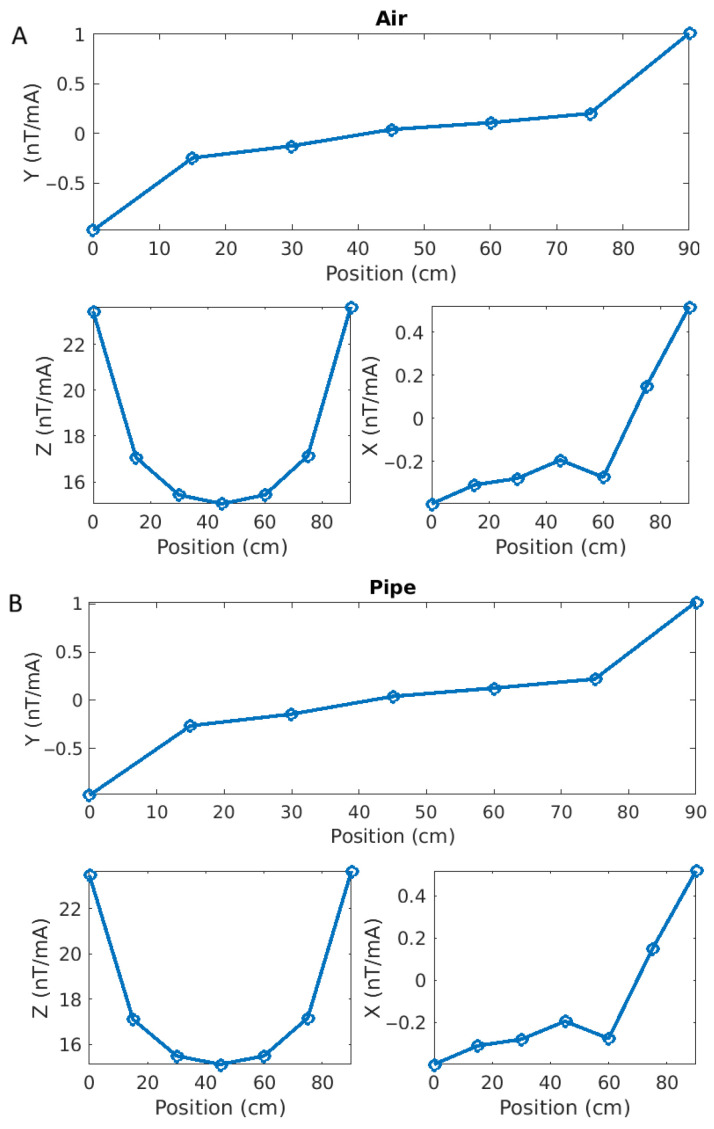
Magnetic responses of the seven sensors for measurements in air (**A**) and over a steel pipe (**B**). The Y- (**top**), X- (**bottom left**) and Z-components (**bottom right**) are normalized to source current.

Figure 25 shows a comparison between two tests: one in air (left) and one with a small steel pipe laid 45 cm under the prototype (right). The Y- (top) and X- and Z-components (bottom) of the magnetic response normalized to current are presented. Each circle denotes the value at one sensor positioned between 0 and 90 cm. There is very little difference between the two cases.

**Figure 26:**
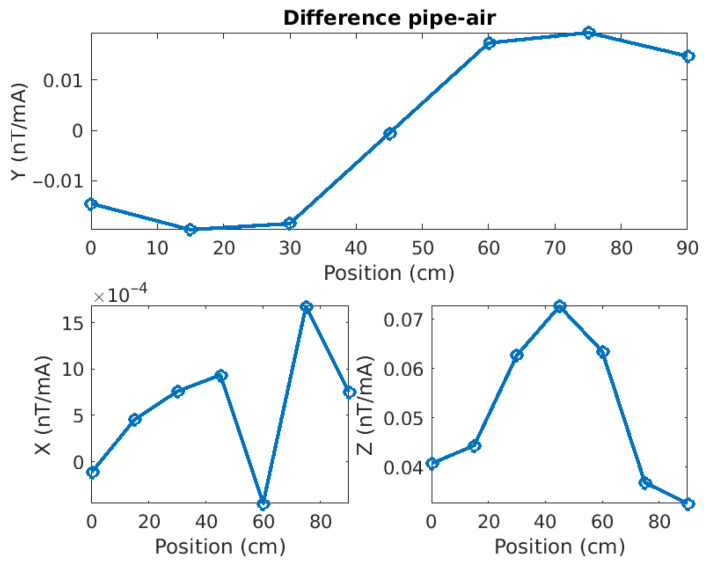
Differences between the magnetic responses for the pipe and the air cases. The shapes are similar to those obtained by numerical modelling, especially for the Y- and Z-components.

However, if we compute the difference between the two responses (here Pipe minus Air, Figure 26), we see that not only are they not the same, but they also show a clear pattern.

- the Y-component anomaly shape corresponds very well with what we obtained by numerical modelling (e.g., Figure 8);
- the Z-component anomaly shows a clear response centered on the pipe axis, again in good agreement with the modelling results;
- the X-component anomaly is much weaker than the two others (10 to 45 times less) and is likely affected by ambient noise. Its inflexion point is near the pipe axis, but the values on the right-hand side (y > 60 cm) seem inconsistent.

## 5. Pipe Defect Detection Experiment

**Figure 27:**
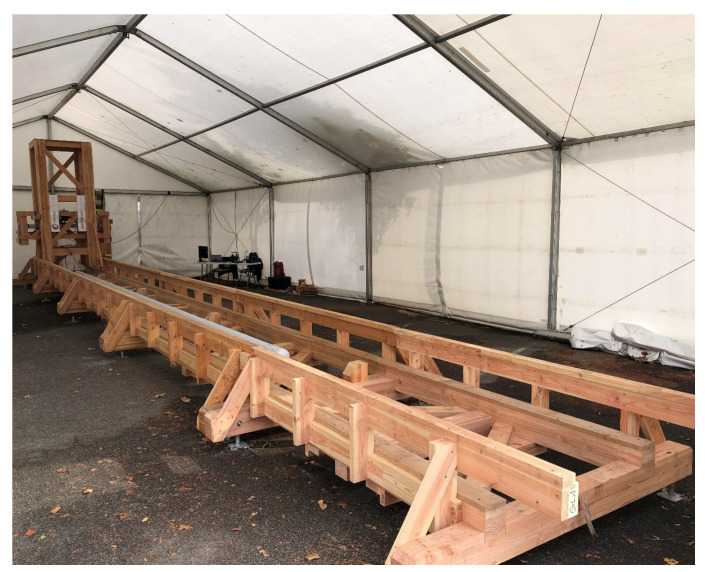
Wooden bench designed by Skipper NDT at the CETIM research facility in Senlis, France.

We ran a first test of the prototype on a custom wooden bench designed by Skipper NDT (Figure 27) and installed at the CETIM research facility in Senlis, France. The test bench is 15 m long. It supports a tower, which is 4 m high and which rolls or slides on wooden tracks along the bench. The tower includes an elevator with a vertical range of 2.3 m in 5 cm increments. This elevator accommodates the instruments: the array of triaxial flux-gate magnetometers and the active-source coil. The support instrumentation, current source and data logger are placed on a movable cart-table next to the tower. The 15 m long bench accommodates pipeline sections of 12 m length. Their typical diameter is 17 cm with an 8 mm wall thickness. The bench is oriented mostly in the east–west direction, deviating from true magnetic east–west by about 7°. The earth magnetic field in Senlis is about 21,700 nT north–south and 44,100 nT in the vertical direction.

### 5.1. Noise Test

**Figure 28:**
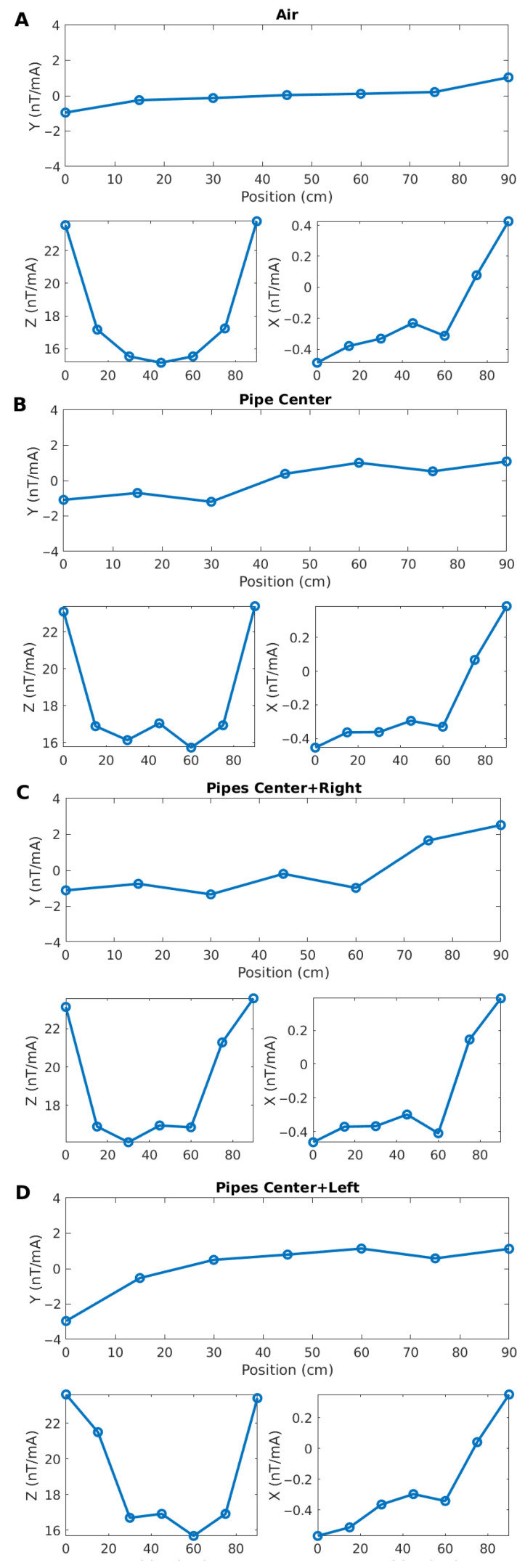
Magnetic field responses on the bench for measurements over air (**A**), over a single piece of pipe centered on the sensor bar (**B**), over two small pipes located under the center and to the right of the bar (**C**) and over two small pipes located under the center and to the left of the bar (**D**). Fields are normalized to the current source.

Before scanning an actual pipe with the prototype, we first tested its response on the bench under local magnetic-noise conditions. We first tested it in air (Figure 28A), then over a single small steel pipe in the center of the sensor bar (Figure 28B), then over two small pipes located under the center and to the right of the bar (Figure 28C) and over two small pipes located under the center and to the left of the bar (Figure 28D).

Because the pipes are located close to the sensors, their impact is immediately visible, even without processing. The effect of including even a single pipe is obvious when comparing the Y and Z components in Figure 28A,B. Similarly, adding a second piece of pipe also has a strong effect, as shown in Figure 28C,D. The shapes of the curves are also consistent with our earlier finite-element computations. However, the effect of adding pipes on the X component is not so clear. This component is, in fact, very small compared to the others and is much more affected by the ambient noise.

This first test confirms that indeed the prototype works as expected. The layout was, however, very favorable: the pipes were only a few cm below the sensors. The next step was to measure under conditions closer to realistic operations.

### 5.2. Scan of a Damaged Pipe

**Figure 29:**
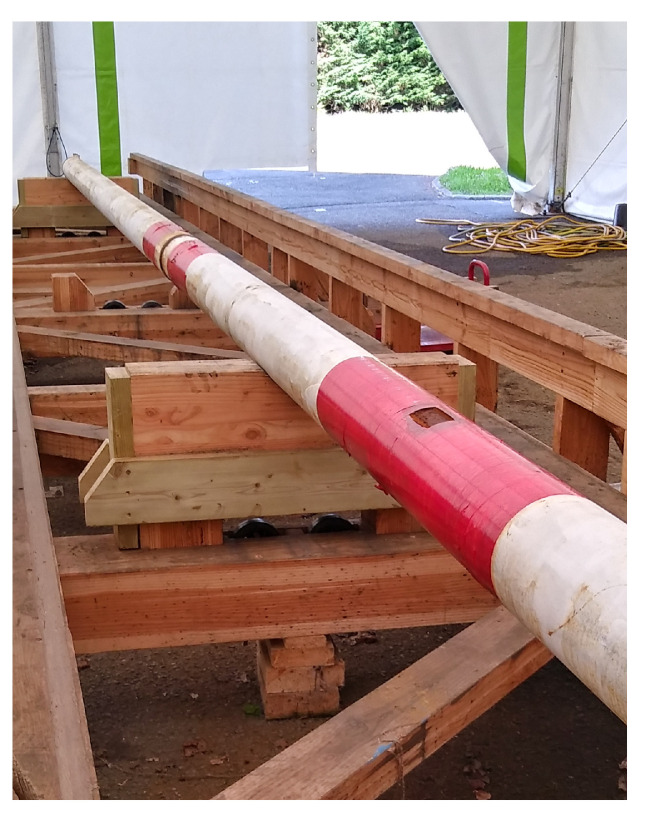
Pipe #40 lying on the measurement bench.

**Figure 30:**
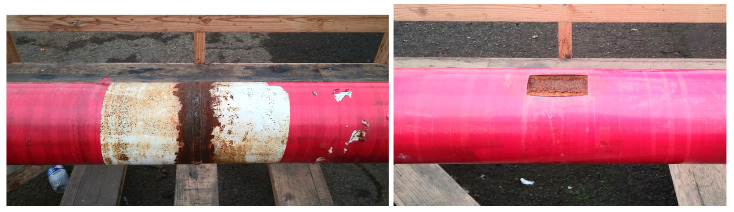
Zoom of the welding joint (**left**) and corrosion (**right**) on Pipe #40.

**Figure 31:**
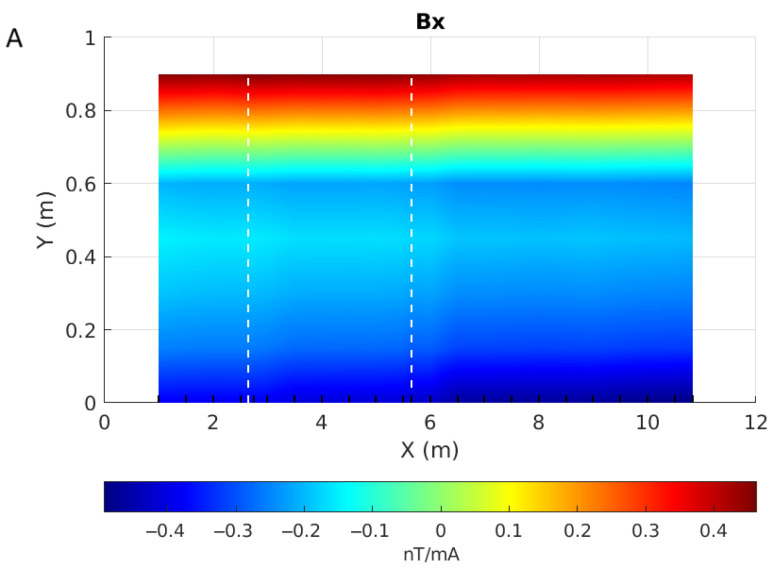

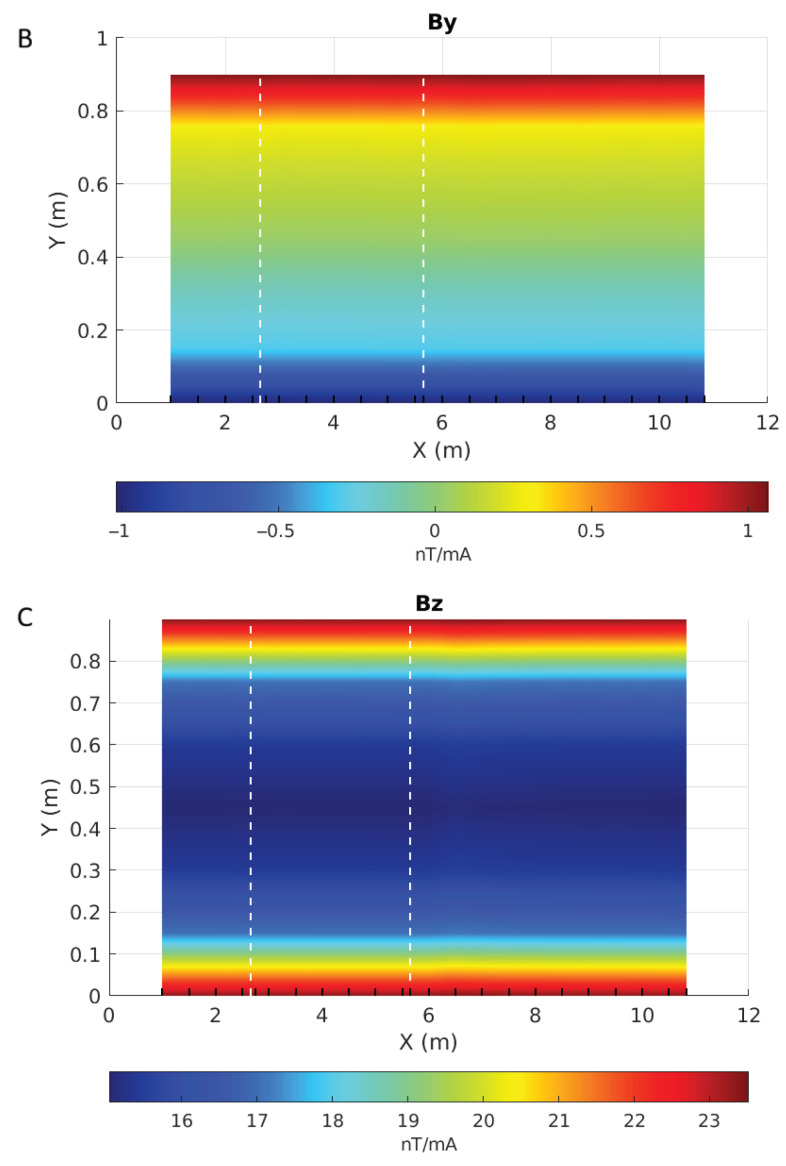
Maps of the magnetic response measured 62 cm above the top of the pipe for components Bx (**A**), By (**B**) and Bz (**C**). X and Y axes are the sensor positions in metres, each line of acquisition is symbolized by a black wedge on the X axis, and white dashed lines give the locations of the anomalies on the pipe. Note that each map has its own colour scale.

We tested our prototype by scanning a test pipe (#40, Figure 29) installed on the wooden bench, 62 cm below the prototype. This pipe has an approximately 10 cm by 4 cm corroded patch at x = 2.75 m as well as a 6 cm-wide welding joint between two sections located at 5.65 m from its origin (Figure 30). We scanned the pipe every 50 cm and densified our measurements near the anomalous areas. Maps of the 3 components of the magnetic responses measured along the pipe, normalized to the injected current, are shown in Figure 31.

**Figure 32:**
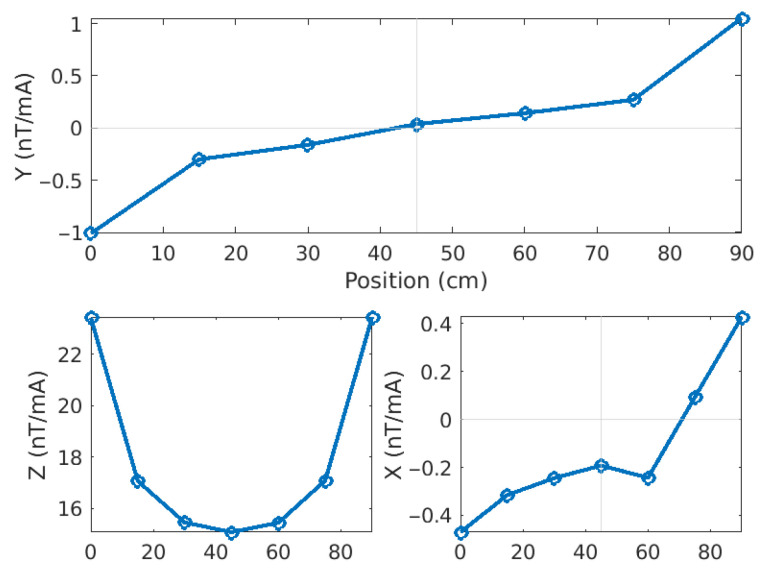
Magnetic field responses measured 62 cm above the top of the pipe for components Bx, By and Bz, along one line of acquisition (x = 8 m). Fields are normalized to current source.

As expected from the above numerical and experimental results, the data in Figure 31 show $B_{z}$ as the strongest and $B_{x}$ as the weakest component. We expect that sensor 4, located in the center of the device at Y = 0.45 m, should correspond to the inflection point of the Bx and By curves, i.e., near 0 nT/mA. This is, however, not the case, according to the maps obtained for these two components. Figure 32 shows the three magnetic components measured along one line of acquisition, for example, highlighting the slight shift of the inflection point on the By curve and the stronger shift on the Bx curve. This shift can be explained by the presence of noise, interfering badly with the small values of Bx, and by sensor 4 not being exactly aligned with the source loop axis.

**Figure 33:**
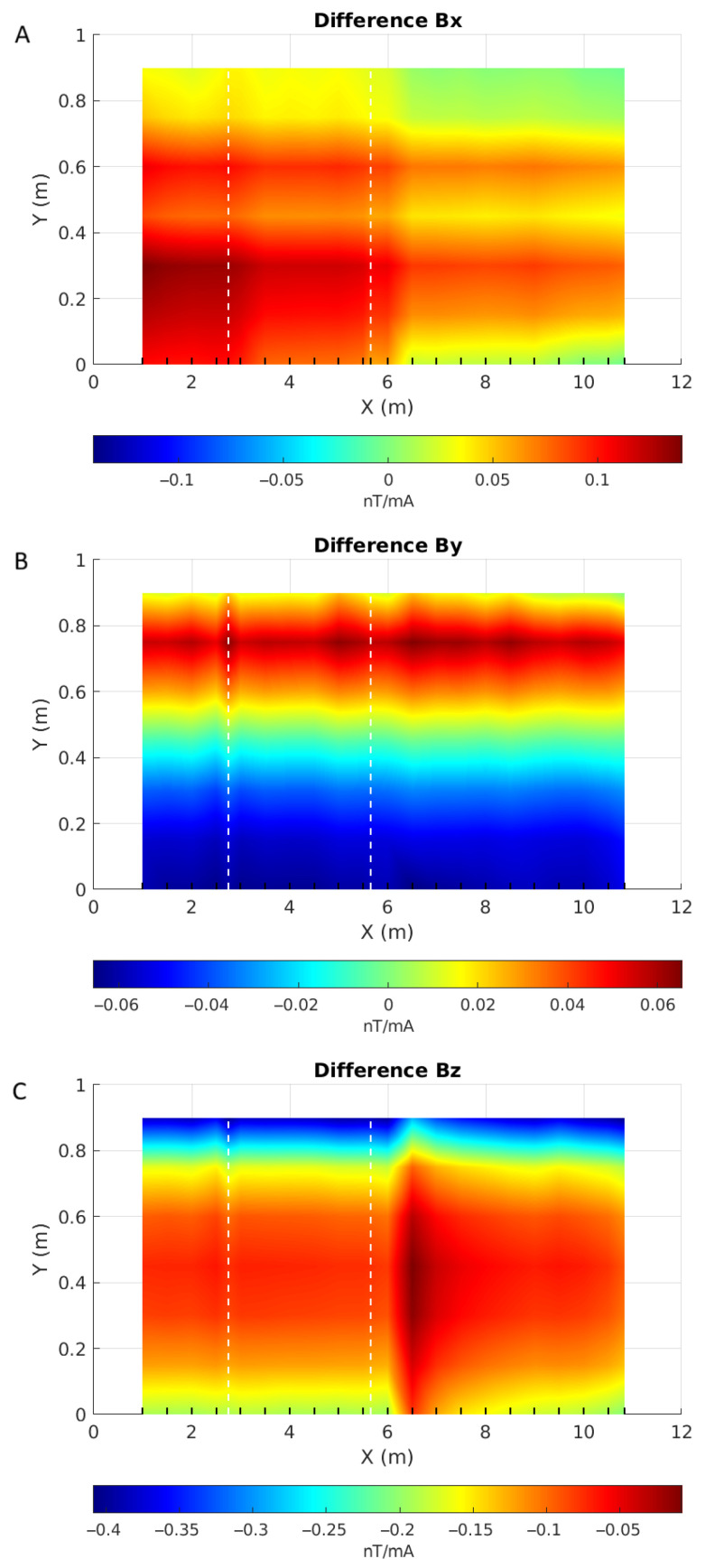
Maps of the differences between components X (**A**), Y (**B**) and Z (**C**) of *B* acquired over a pipe and in air. X and Y axes are the sensor positions in metres, each line of acquisition is symbolized by a black wedge on the X axis, and white dashed lines give the locations of the anomalies on the pipe. Note that each map has its own colour scale.

However, the maps of Figure 31 do not provide much information, as the signal from the source will dominate the responses. To emphasize the anomalous responses, we subtracted from these responses the one acquired over air (Figure 33). These difference maps show that $B_{z}$ has a very strong anomaly to the right of the welding joint at X = 6.5 m. That it does not coincide with the location of the welding joint is not necessarily surprising: welding heats up the steel around it, which will change the distribution of its magnetic properties.

The maps also show a local anomaly in the Y-component that coincides with the location of the corrosion patch. It appears stronger at higher Y values, where the response difference is positive.

**Figure 34:**
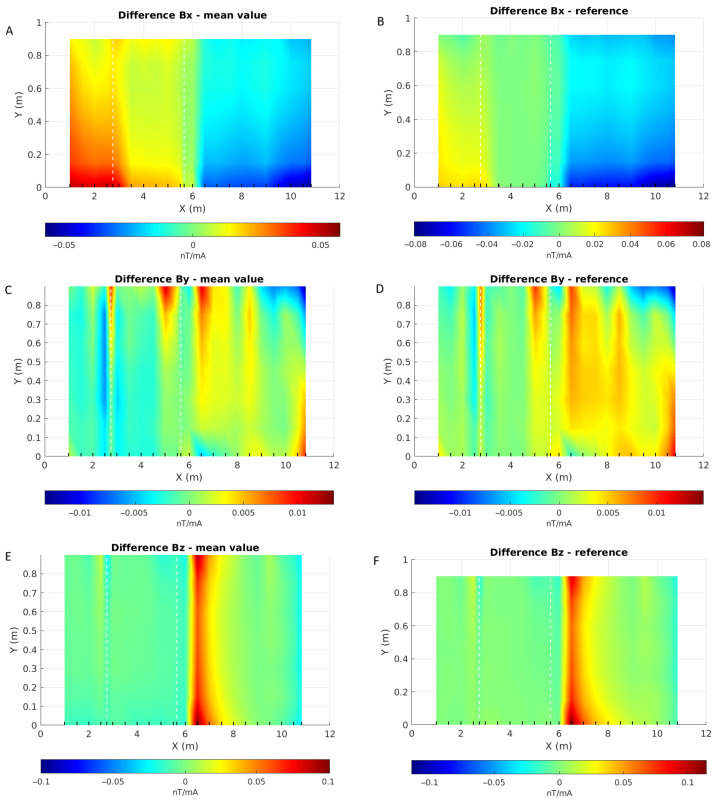
Maps of the differences between each component of *B* acquired over a pipe and the average values (**A**,**C**,**E**) and reference values taken at X = 4 m (**B**,**D**,**F**). X and Y axes are the sensor positions in metres, each line of acquisition is symbolized by a black wedge on the X axis and white dashed lines give the locations of the anomalies on the pipe. Note that each map has its own colour scale.

Another way to emphasize the anomalous responses is to subtract the response from a reference position, i.e., where the pipe shows no damage, or by removing the average values over the length of the pipe (Figure 34). The first observation that jumps to the eye is the partition of the X-component normalized responses: the two pipe sections are clearly delineated, indicating that they have different magnetic properties. The strong anomalies observed near the welding joint on the Z component and near the corrosion on the Y-component are still present. However, one must exert caution when subtracting data acquired at other locations of the pipe.

**Figure 35:**
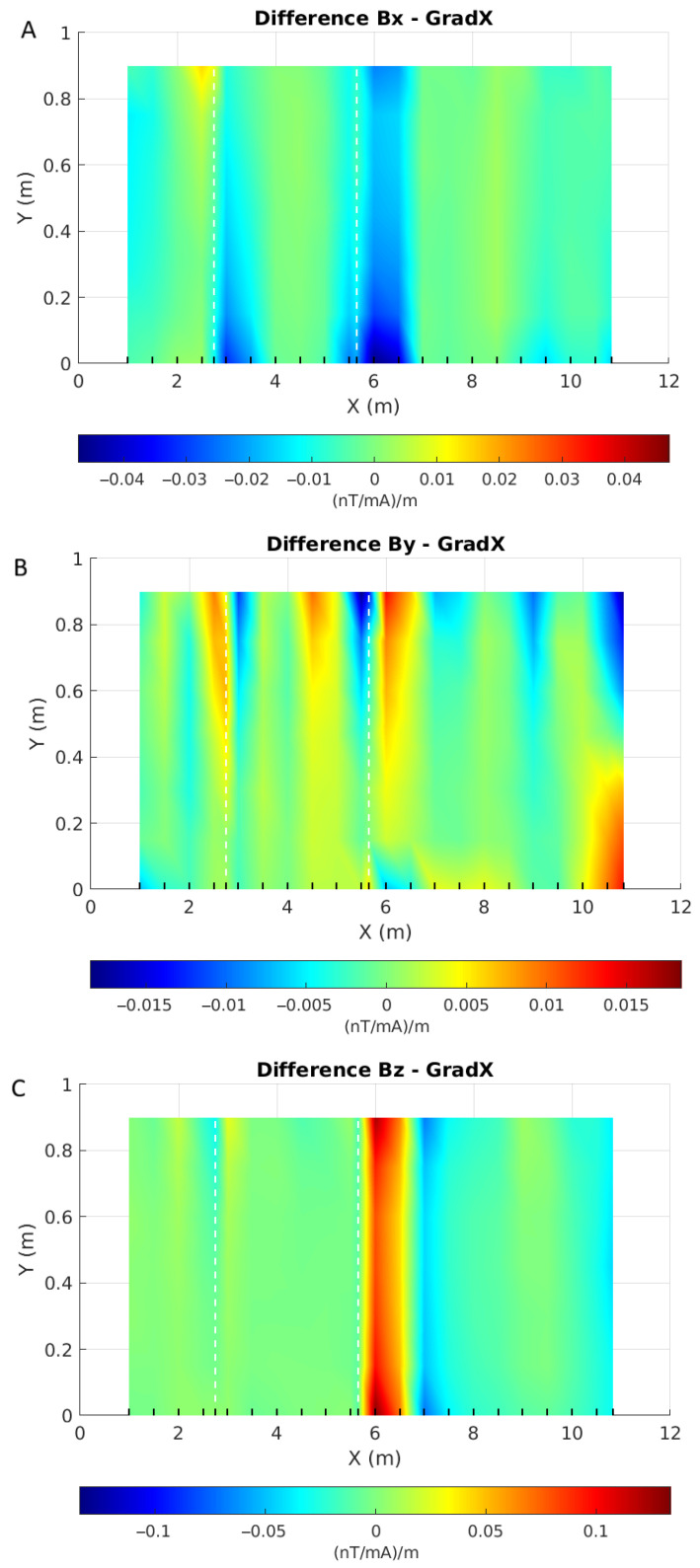
Maps of the gradient (in nT/mA) in the X-direction of components X (**A**), Y (**B**) and Z (**C**) of *B* acquired over the pipe. X and Y axes are the sensor positions in metres, each line of acquisition is symbolized by a black wedge on the X axis, and white dashed lines give the locations of the anomalies on the pipe. Note that each map has its own colour scale.

To get around that difficulty, one can also compute the gradient of the responses. Figure 35 shows this gradient in the direction of the pipe axis. Clear anomalies coincident with the altered areas are now visible, especially on the X- and Z- components. The Y-component also shows some anomalies at the right locations, but also other anomalies along the pipe, which makes their interpretation difficult.

## 6. Discussion

These first results confirm the relevance of using the physics of magnetic induction for the investigation of steel pipes. In this section, we would like to address two major issues: how accurate and reliable the results obtained are and how one can transpose the results obtained on a bench to real-life situations.

### 6.1. Uncertainty Analysis

Quantifying the uncertainties regarding the results is key prior to pushing for further development of the methodology. Should these prove to be large, they would reduce its operational range greatly. Consider a typical acquisition run. We measure the loop source current and 21 (7 3-component sensors) individual magnetic components at 1000 Hz in a noisy environment.

At one end, the source current itself does not behave like an ideal square-wave source. Indeed, the self-inductance and resistance of the loop act as a high-pass filter that deforms the injected square wave when the transmitter current is either switched on or off. Therefore, one needs to remove a few samples around the switching times. In addition, to reduce the effect of offsets on the source current, the positive and negative current periods were separately averaged. These current averages can differ in magnitude by several percent.

At the other end, the magnetic noise sources are multiple: the Earth’s ambient magnetic field, the remanent magnetization of the pipe (and eventually of surrounding rocks), the pipe’s cathodic protection, and other electrically-powered equipment in the vicinity, including the acquisition electronics. These noises are usually either DC or high-frequency, but any long-period noise that varies within a measurement sequence can be problematic.

A simple sum or difference will not always properly decouple the signals from the external magnetic inductions (including the earth’s field and local magnetic field sources) $B_{e}$ and the magnetic induction due to the loop $B_{\ell }$. A simple weight of the measured signals by the current will not ensure the mutual decoupling of these inductions.

Instead, the data sets for positive and negative currents must be treated as a system of two equations with two unknowns. Remembering that the magnetic induction due to the loop $B_{\ell }$ is proportional to the current in the loop:

(5) $$B_{\ell }=\frac{\partial B_{\ell }}{\partial I}I$$

The derivative is constant (from Biot-Savart’s Law) and specific to each component. The measured induction $B_{+}$ for positive current $I_{+}$ and $B_{-}$ for negative current $I_{-}$ are the superposition:

(6) $$B_{+}=B_{e}+\frac{\partial B_{\ell }}{\partial I}I_{+}$$

(7) $$B_{-}=B_{e}+\frac{\partial B_{\ell }}{\partial I}I_{-}.$$

This system of two equations in two unknowns is written in matrix-vector form:

(8) $$\left(\begin{array}{c} B_{+}\\ B_{-} \end{array}\right)=\left(\begin{array}{cc} 1 & I_{+}\\ 1 & I_{-} \end{array}\right)\left(\begin{array}{c} B_{e}\\ \partial B_{\ell }/\partial I \end{array}\right)$$

The matrix is inverted providing $B_{e}$ and $B_{\ell }$:

(9) $$\left(\begin{array}{c} B_{e}\\ \partial B_{\ell }/\partial I \end{array}\right)=\frac{1}{I_{-}-I_{+}}\left(\begin{array}{cc} I_{-} & -I_{+}\\ -1 & 1 \end{array}\right)\left(\begin{array}{c} B_{+}\\ B_{-} \end{array}\right)$$

Explicitly, $B_{e}$ and $B_{\ell }$ are given as separate equations in terms of the measured inductions $B_{+}$ and $B_{-}$.

(10) $$B_{e}=\frac{I_{-}B_{+}-I_{+}B_{-}}{I_{-}-I_{+}}$$

(11) $$\frac{\partial B_{\ell }}{\partial I}=\frac{B_{-}-B_{+}}{I_{-}-I_{+}}.$$

The induction derivative is converted back to the induction $B_{\ell }$ by formally multiplying with an I=1A normalized current. The resulting values for the 3 × 7 measured magnetometer components are entered into the inversion and sensitivity analysis.

For each acquisition cycle, we can estimate the uncertainties on the data by splitting the time series into its positive and negative current intervals. Hence, the current and all magnetic components are separately averaged; their standard deviations ΔI and ΔB provide the error estimate. These standard deviations are square added, assuming their statistical independence. The uncertainties in $B_{e}$ and $B_{\ell }$ are propagated from the errors of the current and induction-component measurements. This error propagation gives the earth magnetic induction

(12) $$\Delta B_{e}=\sqrt{{\left(\frac{\partial B_{e}}{\partial I_{-}}\right)}^{2}\Delta I_{-}^{2}+{\left(\frac{\partial B_{e}}{\partial I_{+}}\right)}^{2}\Delta I_{+}^{2}+{\left(\frac{\partial B_{e}}{\partial B_{-}}\right)}^{2}\Delta B_{-}^{2}+{\left(\frac{\partial B_{e}}{\partial B_{+}}\right)}^{2}\Delta B_{+}^{2}}$$

or

(13) $$\Delta B_{e}=\sqrt{\frac{I_{+}^{2}{\left(B_{-}-B_{+}\right)}^{2}}{{\left(I_{-}-I_{+}\right)}^{4}}\Delta I_{-}^{2}+\frac{I_{-}^{2}{\left(B_{+}-B_{-}\right)}^{2}}{{\left(I_{-}-I_{+}\right)}^{4}}\Delta I_{+}^{2}+\frac{I_{+}^{2}\Delta B_{-}^{2}+I_{-}^{2}\Delta B_{+}^{2}}{{\left(I_{-}-I_{+}\right)}^{2}.}}$$

For the induction due to the loop,

(14) $$\Delta B_{\ell }=I\sqrt{{\left(\frac{\partial \left(\frac{\partial B_{c}}{\partial I}\right)B_{e}}{\partial I_{-}}\right)}^{2}\Delta I_{-}^{2}+{\left(\frac{\partial \left(\frac{\partial B_{c}}{\partial I}\right)B_{e}}{\partial I_{+}}\right)}^{2}\Delta I_{+}^{2}+{\left(\frac{\partial \left(\frac{\partial B_{c}}{\partial I}\right)B_{e}}{\partial B_{-}}\right)}^{2}\Delta B_{-}^{2}+{\left(\frac{\partial \left(\frac{\partial B_{c}}{\partial I}\right)B_{e}}{\partial B_{+}}\right)}^{2}\Delta B_{+}^{2}}$$

or

(15) $$\Delta B_{\ell }=I\sqrt{\frac{{\left(B_{+}-B_{-}\right)}^{2}}{{\left(I_{-}-I_{+}\right)}^{4}}\left(\Delta I_{-}^{2}+\Delta I_{+}^{2}\right)+\frac{B_{-}^{2}+B_{+}^{2}}{{\left(I_{-}-I_{+}\right)}^{2}.}}$$

**Figure 36:**
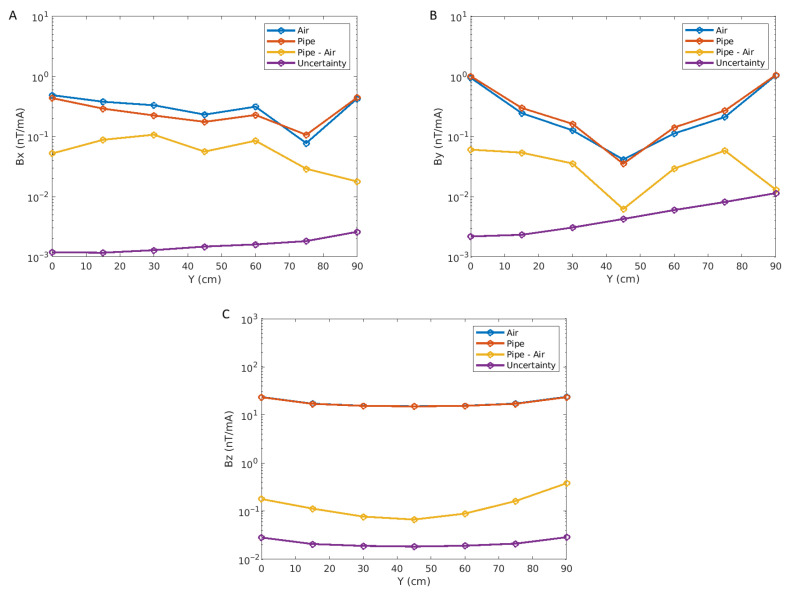
Plots of the X (**A**), Y (**B**) and Z (**C**) components of the magnetic fields acquired in air (blue), averaged over the pipe (orange), averaged over the pipe minus in air (yellow) and uncertainty on the average over the pipe for each sensor (purple). The x-axis denotes the sensor position along the central bar of the acquisition in our prototype.

We used the above approach to compute uncertainties regarding measurement data. Figure 36 shows a comparison of measurements in air and above the pipe, the difference between them, and the computed uncertainty. Most uncertainty values are very small: around $10^{-3}$ nT/mA for the X component, less than $10^{-2}$ nT/mA for the Y component and less than $3\times 10^{-3}$ nT/mA for the Z component, i.e., much smaller than their respective *B* values.

**Figure 37:**
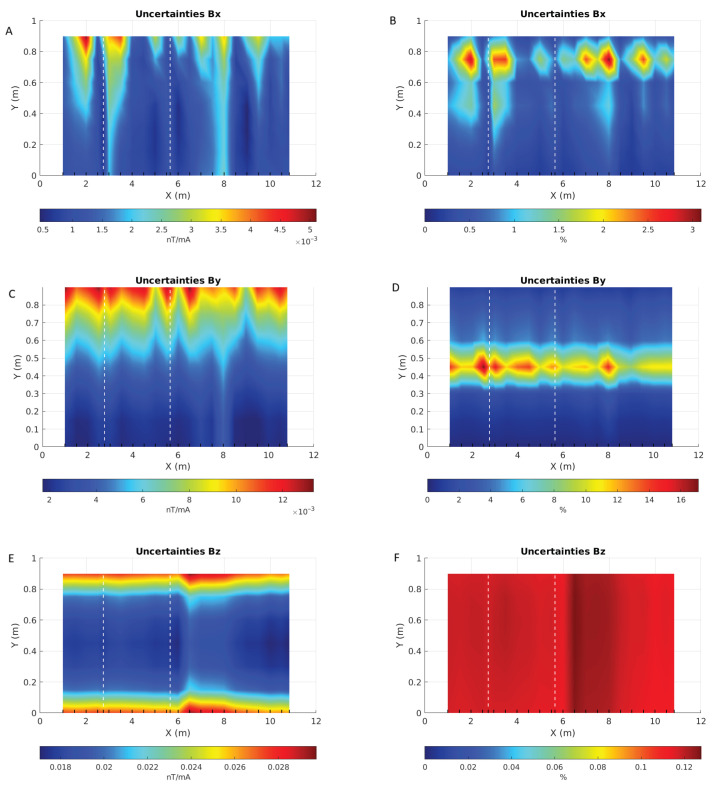
Maps of the absolute (in nT/mA, (**A**,**C**,**E**)) and relative (in %, (**B**,**D**,**F**)) uncertainties for each component of *B* acquired over the pipe. X and Y axes are the sensor positions in meters. Note that each map has its own colour scale.

Figure 37 shows a comparison of the absolute (left column) and relative (right column) uncertainties for all components. Relative errors are overall very small, except where the *B* values themselves are near zero. Neglecting these very small values, errors are mostly less than 1 percent for the X component, less than 4 percent for the Y component and less than 0.1 percent for the Z component.

Other sources of uncertainty that we have not analyzed include the orthogonality of the three magnetic components for each sensor and their proper alignment and location on the central bar. We have no control over the former, as it depends on the sensor manufacturer. We could act on the latter by manufacturing the prototype ourselves if these issues arise.

### 6.2. Toward Operational Use Cases

These first results on a test bench demonstrate that an active magnetic induction system can successfully localize corrosion-related damages as well as welding joints on steel pipes. Our active method does not allow us to distinguish between these two features. We have observed, however, that welding joints show a contrast in remanent magnetization that can be measured when the pipe is not excited by the source, i.e., by doing a passive magnetic measurement. We can also see a remanence effect in the difference maps for the X component (Figure 34A,B). This is somehow expected as the pipe response is much weaker for this component.

The next logical step is to consider what possible problems need to be addressed before deploying it in an operational setting.

For the case of subsurface pipes, the first difficulty arises from the depth of burial. For our 1.2 m × 0.75 m source loop prototype, we simulated a pipe buried at a depth of 0.62 m. In reality, pipes are often buried at greater depths, so a larger source loop is required to ensure proper penetration of the inducing field. More magnetic sensors are also required to properly sample the magnetic responses. Our modelling results in Section 3.1 show that a 4 m × 1 m source loop is sufficient to induce magnetization at a few metres depths.

Our bench test also neglects the presence of geological materials between the instrument and the target pipe. Our results are most likely valid for a pipe recovered by fine-grained sands. However, the presence of pebbles or coarser rocks could pose a challenge: they often contain iron oxides that can be magnetized by the source loop. They have obviously much lower magnetic permeabilities than steel but they are located closer to the device (field amplitude decreases roughly as $1/r^{3}$) and so may add significant “geological noise” to the measured data.

Of course, the above challenges are not relevant to pipes above the surface or lying on the ocean floor. In these cases, perturbations due to the instrument carrier will pose greater challenges.

## 7. Conclusions

We have demonstrated, first by numerical modelling, then by an experiment on a bench, the feasibility of investigating damages on steel pipes with an active magnetic induction-based device. Our test results show that both corrosion and welding joints are readily identified after a simple processing workflow. The methodology, however, still has some ambiguities when it comes to differentiating between magnetic permeability anomalies related to corrosion and those related to welding joints. Processing the magnetic signals separately when the source is active and inactive will help determine the source of the observed anomalies. In all cases, however, 3-component magnetic sensors are required as components behave differently to these anomalies. As the most reliable interpretation is based on spatial derivatives of the observed magnetic fields, a larger number of magnetic sensors oriented perpendicular to the pipe and a tighter spacing of sampling along the pipe are required.

## Figures and Tables

**Table 1:** Electrical and magnetic properties of selected materials. From Bozorth, 1993 [12].

Material	σ (S/m)	$\mu_{r}$ (SI)
Iron	$10\times 10^{6}$	150
Steel (0.001% C)	$10\times 10^{6}$	4400
Steel (0.02% C)	$10\times 10^{6}$	400
Steel (0.2% C)	$10\times 10^{6}$	120
1040 Alloy	$5\times 10^{6}$	40,000

**Table 2:** Dimensions of the objects that constitute the different modeling geometries.

	**X Dimension**	**Y Dimension**	**Z Dimension**
Loop n1	1 m	2 m	15 cm
Loop n2	1 m	3 m	15 cm
Loop n3	1 m	4 m	15 cm
	**Length**	**Outer Radius**	**Inner Radius**
DN150	15 m	0.08415 m	0.07704 m
DN200	15 m	0.10950 m	0.10132 m
DN400	15 m	0.20320 m	0.19367 m

**Table 3:** Physical properties of materials mentioned for initial modeling.

	Rel. Permeability	Conductivity	Rel. Permittivity
	$\mu_{r}$	σ	$\epsilon_{r}$
Air	1	$10^{-5}$	1
Ground	1	0.1	1
Steel	100	$10^{6}$	1

**Table 4:** Summary of the numerical simulation results.

Perturbation	Impact on Peak B wrt Clean Pipe			
	Casing	20 cm Anomaly	60 cm Anomaly	100 cm Anomaly
$\mu_{r}$/2	35%			
σ±10%	none			
Section $\mu_{r}$/10		2.4%	6.6%	15.0%
Section $\mu_{r}$/100		4.3%		
Half-section $\mu_{r}$/10		1.8%		
Half-section $\mu_{r}$/100		3.0%		

## Data Availability

The datasets presented in this article are not readily available due to technical limitations. Requests to access the datasets should be directed to the corresponding author.
